# RETICULATA1 is a plastid-localized basic amino acid transporter

**DOI:** 10.1038/s41477-025-02080-z

**Published:** 2025-08-22

**Authors:** Franziska Kuhnert, Philipp Westhoff, Vanessa Valencia, Stephan Krüger, Karolina Vogel, Peter K. Lundquist, Christian Rosar, Tatjana Goss, Andreas P. M. Weber

**Affiliations:** 1 Institute of Plant Biochemistry, Heinrich Heine University, Düsseldorf, Germany; 2 Metabolomics and Metabolism Laboratory, Heinrich Heine University, Düsseldorf, Germany; 3 Cluster of Excellences in Plant Science (CEPLAS), Heinrich Heine University, Düsseldorf, Germany; 4 Present Address: Plant Energy Biology, University of Western Australia, Crawley, Western Australia Australia; 5 Present Address: Department of Biochemistry and Molecular Biology, Michigan State University, East Lansing, MI USA

**Keywords:** Plant physiology, Plant transporters

## Abstract

Plants have a crucial role in providing essential amino acids for human nutrition. Nine of the 20 proteinogenic amino acids are exclusively synthesized de novo in plastids, yet transporters mediating their exchange across the plastid inner envelope remain unknown. Here we identify RETICULATA1 (RE1) as a plastid-localized transporter for basic amino acids—including Arg, Citr, Orn and Lys—in *Arabidopsis thaliana*. Loss-of-function mutants display a reticulate leaf phenotype, contain lower amounts of basic amino acids and are impaired in amino acid homeostasis. RE1 belongs to a novel class of membrane transport proteins that contain a domain of unknown function 3411 and are found exclusively in plastid-containing organisms. Our results indicate functional overlap with its closest homologue RER1, as the double mutant is lethal. Isotope labelling reveals that loss of *RE1* reduces basic amino acid biosynthesis and affects the equilibration of plastidic and cytosolic amino acid pools. These findings uncover a critical role for plastidial amino acid transporters in coordinating primary metabolism, development and nutrient allocation in plants.

## Abstract (web summary)

RETICULATA1 is a plastid membrane transporter in *Arabidopsis* that enables basic amino acid exchange across the plastid inner envelope. Loss-of-function mutants reveal its essential role in amino acid homeostasis, plant development and seed production.

## Main

Amino acids are essential building blocks for all forms of life on Earth. They constitute proteins and participate in numerous metabolic reactions in plant cells. Given their pivotal roles in plant metabolism, the biosynthesis, degradation, storage and distribution of amino acids must be strictly regulated^1–3^. Unlike most eukaryotes, plants can synthesize all 20 proteinogenic amino acids de novo. Within a plant cell, amino acid biosynthesis is compartmentalized: the essential amino acids His, Ile, Leu, Lys, Thr, Trp and Val, as well as the non-essential amino acids Arg and Tyr, are synthesized exclusively in the plastids^3,4^. Over the past decades, substantial progress has been made in identifying the genes encoding soluble enzymes of the plastidial amino acid biosynthetic pathways, and in deciphering their feedback regulatory mechanisms^3,5–7^. Yet, despite this progress, no plastidial transporter for shuttling any of these nine amino acids across the plastid inner envelope has been identified in plants so far.

During plastid biogenesis, the nascent plastid must be supplied with amino acids from the cytoplasm for protein biosynthesis. Once plastids are established, amino acids synthesized in plastids, as well as those released by protein degradation^8^, must be exported. Hence, transporters that catalyse bidirectional amino acid transport across the plastid envelope are essential. Despite their importance, our understanding of intracellular amino acid transport remains limited. So far, only three plastidial amino acid transporters have been characterized: the DICARBOXYLATE TRANSPORTER 2.1 (DiT2.1) from *Arabidopsis thaliana* (*Arabidopsis*), the USUALLY MULTIPLE ACIDS MOVE IN AND OUT TRANSPORTER 44 (UMAMIT44) from *Arabidopsis* and the cationic amino acid transporter (CAT) from *Petunia hybrida* (*Petunia*). DiT2.1 exports Glu in exchange for malate and plays a crucial role in de novo nitrogen assimilation and ammonia refixation during photorespiration^9,10^. UMAMIT44, recently identified as a plastidial Glu exporter, maintains cellular Glu homeostasis and influences nitrogen partitioning to sink tissues in *Arabidopsis*^11^. *Petunia* CAT exports Phe from plastids and contributes to the regulation of aromatic amino acid metabolism^12^. *Arabidopsis* homologues of the *Petunia* CAT with predicted plastid transit peptides have yet to be characterized. Although it has been widely accepted that Phe is synthesized exclusively in the plastids, recent evidence suggests that a cytosolic route for Phe biosynthesis may also exist^13–15^.

The basic amino acids Arg and Lys are among the nine amino acids synthesized exclusively in plastids in plants. Lys, an essential amino acid for human nutrition, is synthesized from Asp alongside Met, Ile and Thr^5^. Arg, which has the highest nitrogen-to-carbon ratio among the proteinogenic amino acids, is synthesized from Glu via the intermediates ornithine (Orn) and citrulline (Citr)^4^. In addition to de novo biosynthesis, plants can take up amino acids from the rhizosphere^16,17^. Root uptake and long-distance transport of basic amino acids is largely mediated by the plasma membrane-localized transporters LHT1 and AAP5^18,19^. However, intracellular transport processes for basic amino acids remain poorly understood. Mitochondrial import of basic amino acids is facilitated by the basic amino acid carriers BAC1 and BAC2^20,21^, but the identity of plastidial basic amino acid transporters remains unknown.

RETICULATA1 (RE1) is a member of the RETICULATA (RE) protein family, which comprises eight plastid-localized membrane proteins in *Arabidopsis*. The family is named after the prominent reticulate leaf phenotype observed in the *RE1*-knockout mutant. Although *RE1* was the first reticulate leaf mutant identified in *Arabidopsis* and has been used as a genetic marker for decades, its molecular function has remained unknown^22,23^. Based on its predicted localization, its phenotype, its co-expression with amino acid biosynthetic genes and a proposed role in maintaining amino acid homeostasis^23^, we hypothesized a role in amino acid transport across the plastid envelope. Here, we show that RE1 and its closest homologue RETICULATA-RELATED1 (RER1) function as plastidial basic amino acid carriers in *Arabidopsis*. Using a combination of yeast complementation and functional transport assays, we demonstrate that RE1 mediates the transport of Arg, Lys, Orn and Citr in vitro. Loss-of-function mutants of *RE1* in *Arabidopsis* display reduced basic amino acid content in leaves and isolated chloroplasts, and are impaired in de novo biosynthesis of basic amino acids. These data provide crucial insight into the complex connection between plastidial amino acid transport and leaf development.

## Results

### Identification of plastid-localized amino acid transporter candidates

To identify candidate genes encoding for plastidial amino acid transporter proteins, we searched for genes encoding transmembrane proteins with a plastidial targeting sequence that are co-expressed with genes involved in the de novo biosynthesis of amino acids that are synthesized exclusively in the plastids. Within this co-expression network, we found a gene encoding for a protein with a domain of unknown function 3411 (DUF3411) named *RE1*. *RE1* is co-expressed with genes encoding for proteins involved in Arg and Lys de novo biosynthesis, such as argininosuccinate lyase or diaminopimelate decarboxylases (Supplementary Tables 1–3).

**Fig. 1:**
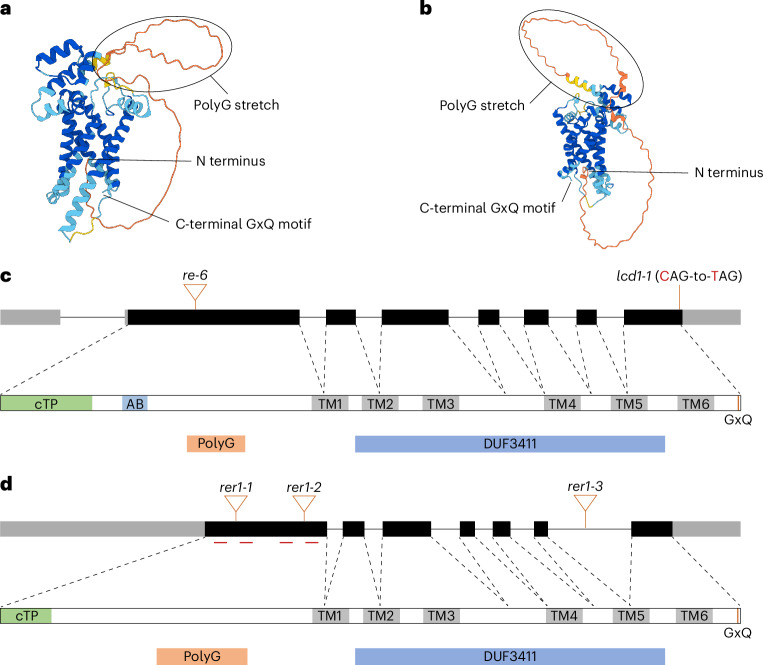
Features and domains of RE1 and RER1. **a**,**b**, The predicted structure of the mature forms of RE1 (**a**) and RER1 (**b**). Structures were predicted using AlphaFold 3^24^. **c**,**d**, Schematic representations of the *RE1* (**c**) and *RER1* (**d**) locus and protein sequence with position of the T-DNA insertion lines, the used guides for the CRISPR–Cas9 approach, the predicted plastid transit peptide (green), the sequence used to obtain the anti-RE1 antibody (light blue) and the predicted transmembrane helices (grey). Exons are depicted as black boxes, and the 5’-untranslated region (UTR) and 3’-UTR are depicted as grey boxes. The relative positions of the polyglycine (polyG) stretch and the domain of unknown function (DUF) 3411 are highlighted in orange and blue, respectively.

**Extended Data Fig. 1:**
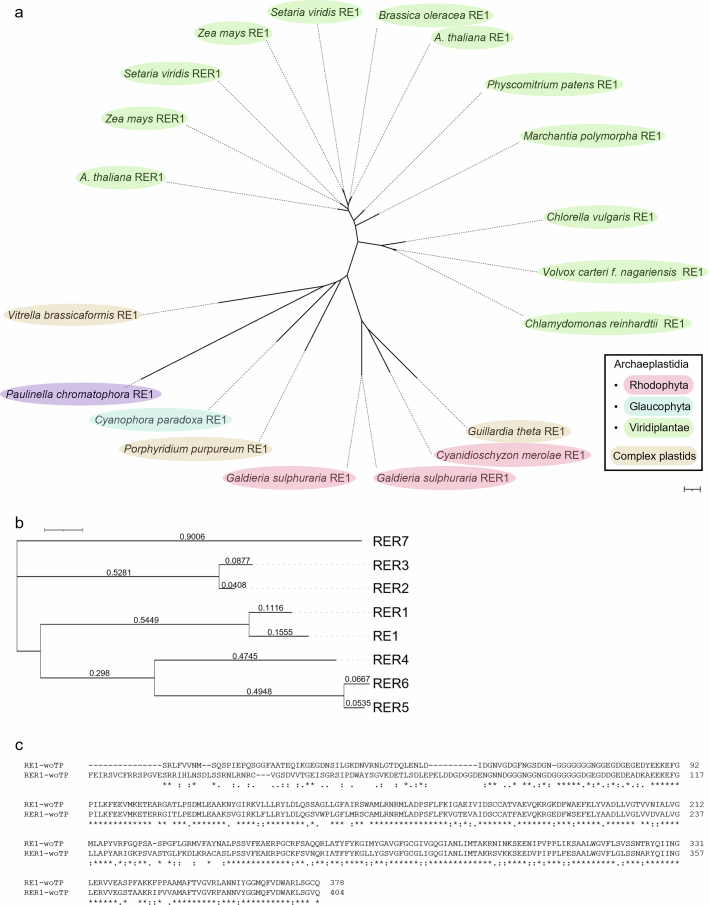
Phylogenetic relationship of RE proteins. (**a**) Neighbor joining tree of RE1 and RER1 orthologues. Tree scale: 0.1. Accession numbers: *A. thaliana* RE1, B9DFK5.1; *A. thaliana* RER1, Q9FGP9.1; *Brassica oleracea*, VDD05637.1; *Chlamydomonas reinhardtii*, XP_001694960.1; *Chlorella vulgaris*, KAI3433810.1; *Cyanidioschyzon merolae*, XP_005537120.1; *Cyanophora paradoxa*, tig00000269_g23772.t1; *Galdieria sulphuraria* RE1, GJD11306.1; *Galdieria sulphuraria* RER1, XP_005703345.1; *Guillardia theta*, XP_005820097.1; *Marchantia polymorpha*, PTQ44604.1; *Paulinella chromatophora* scaffold4653-size1931 (GEZN00000000.1); *Physcomitrium patens*, XP_024368805.1; *Porphyridium purpureum*, KAA8496794.1; *Setaria viridis* RE1, XP_034582302.1; *Setaria viridis* RER1, XP_034602011.1; *Vitrella brassicaformis* RE1, CEM14832.1; *Volvox carteri f. nagariensis*, XP_002954331.1; *Zea mays* RE1, XP_008670913.1; *Zea mays* RER1, NP_001144215.1. (**b**) Neighbor joining tree of *Arabidopsis* RE1 and RER protein sequences with branch lengths. Tree scale 0.1. *Arabidopsis* identifier: AT2G37860 (RE1), AT5G22790 (RER1), AT3G08630 (RER2), AT3G08640 (RER3), AT5G12470 (RER4), AT2G40400 (RER5), AT3G56140 (RER6), AT5G24690 (RER7). Alignments were performed using MAFFT version 7^60^. Substitution model: Jones-Taylor-Thornton. Bootstrap value 100. Trees were constructed using iTOL v7^61^. (**c**) Alignment of mature protein sequences of RE1 and RER1.

**Extended Data Fig. 2:**
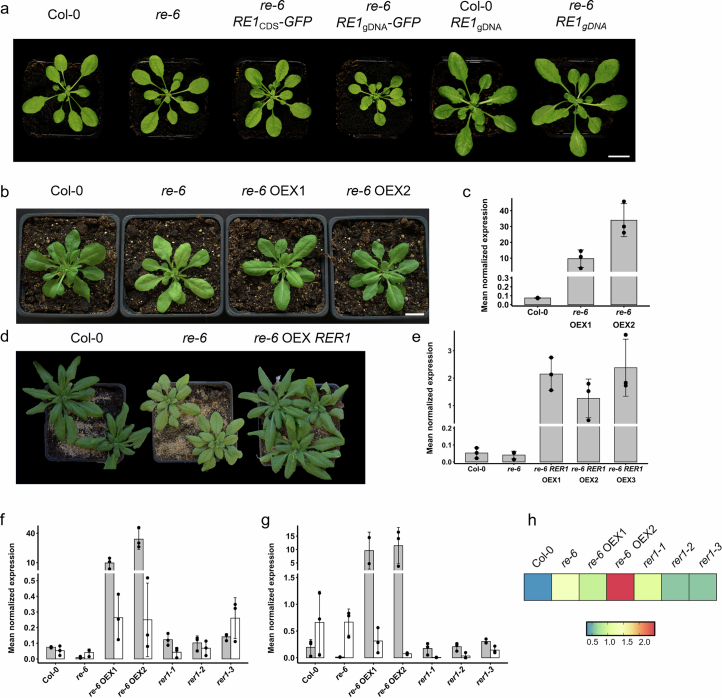
Phenotypes of OEX and expression analysis of RE1 and RER1. (**a**) Representative pictures of 5-week-old Col-0, *re-6* and complementation lines expressing the coding sequence of *RE1* (*RE1*_CDS_) or the genomic DNA of *RE1* (*RE1*_gDNA_) with or without C-terminal GFP fusion in Col-0 or the *re-6* mutant background. Scale bar = 2 cm. (**b**) Representative pictures of 5-week-old Col-0, *re-6, re-6* OEX1 and *re-6* OEX2 plants (from left to right). Scale bar = 2 cm. (**c**) Relative *RE1* transcript levels in 14-d-old Col-0, *re-6* OEX1 and *re-6* OEX2 seedlings. Data are shown as mean ± SD of three biological replicates. (**d**) Representative pictures of 7-week-old Col-0, *re-6, re-6* OEX *RER1* plants (from left to right). Scale bar = 2 cm. (**e**) Relative *RER1* transcript levels in 14-d-old Col-0, *re-6*, *re-6 RER1* OEX1, *re-6 RER1* OEX2 and *re-6 RER1* OEX3 seedlings. Data are shown as mean ± SD of three biological replicates. Relative *RE1* (grey bars) and *RER1* (white bars) transcript levels in (**f**) 14-d-old seedlings and (**g**) 34-d-old Col-0, *re-6*, *rer1-1*, *rer1-2*, *rer1-3*, *re-6* OEX1 and *re-6* OEX2 rosette leaves. Data are shown as mean ± SD of three biological replicates. (**h**) Transcript changes of RE1 in Col-0, *re-6*, *rer1-1*, *rer1-2*, *rer1-3*, *re-6* OEX1 and *re-6* OEX2 shoots versus root tissue. Data are shown as log_2_ fold change of shoot versus root tissue. Source data

RE proteins are encoded exclusively in genomes of organisms that contain plastids (Extended Data Fig. 1a and Supplementary Text). The *Arabidopsis* genome encodes for eight proteins containing a DUF3411: RE1 and its homologues RER1–RER7 (Extended Data Fig. 1b). Among these, RER1 is the closest homologue of RE1, with the mature proteins sharing 65% sequence identity (Extended Data Fig. 1c), raising the possibility of functional similarity. To gain insight into the structural organization of RE1 and RER1, we analysed their predicted structures (omitting the plastid transit peptides) using AlphaFold 3^24^. Both proteins are approximately 40 kDa in size and are predicted to contain four to six transmembrane domains, consistent with their proposed role as membrane transporters. The predicted topology covers the conserved domain of unknown function DUF3411 and includes a highly conserved C-terminal GxQ motif, which is essential for the function of the proteins and prevents C-terminal tagging (Extended Data Fig. 2a and Supplementary Text). In addition, both RE1 and RER1 possess a polyglycine stretch at their N terminus (Fig. 1a–d), a feature conserved across RER proteins predicted to localize to the inner plastid envelope.

**Fig. 2:**
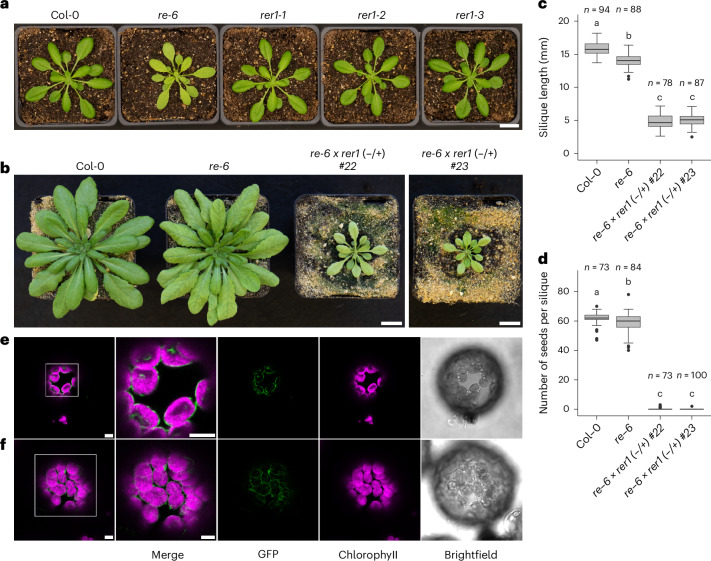
Phenotype of *re-6*-knockout mutants and subcellular localization of RE1 and RER1. **a**, Photos of 4-week-old Col-0, *re-6*, *rer1-1*, *rer1-2* and *rer1-3* plants. Scale bar, 2 cm. **b**, Photos of 7-week-old Col-0, *re-6* and *re-6 × rer1* (−/+) plants. Scale bars, 2 cm. **c**,**d**, Length of mature siliques (**c**) and number of seeds per mature silique (**d**) harvested from Col-0, *re-6* and *re-6 × rer1* (−/+) plants. Data are presented as box and whiskers (minimum to maximum) of at least 78 biological replicates. Different letters indicate statistically significant differences between means (*P* < 0.05; one-way analysis of variance (ANOVA) with Tukey’s test). The box plots show the median (horizontal line), interquartile range (box) and whiskers extending to 1.5× the interquartile range. Exact *P* values are shown in source data with 95% confidence intervals. **e**,**f**, Localization of RE1 (**e**) and RER1 (**f**) GFP fusion constructs (green) with chlorophyll autofluorescence (magenta) in *Nicotiana benthamiana* protoplasts isolated 2 days after infiltration. Scale bars, 5 µm. Source data

**Extended Data Fig. 3:**
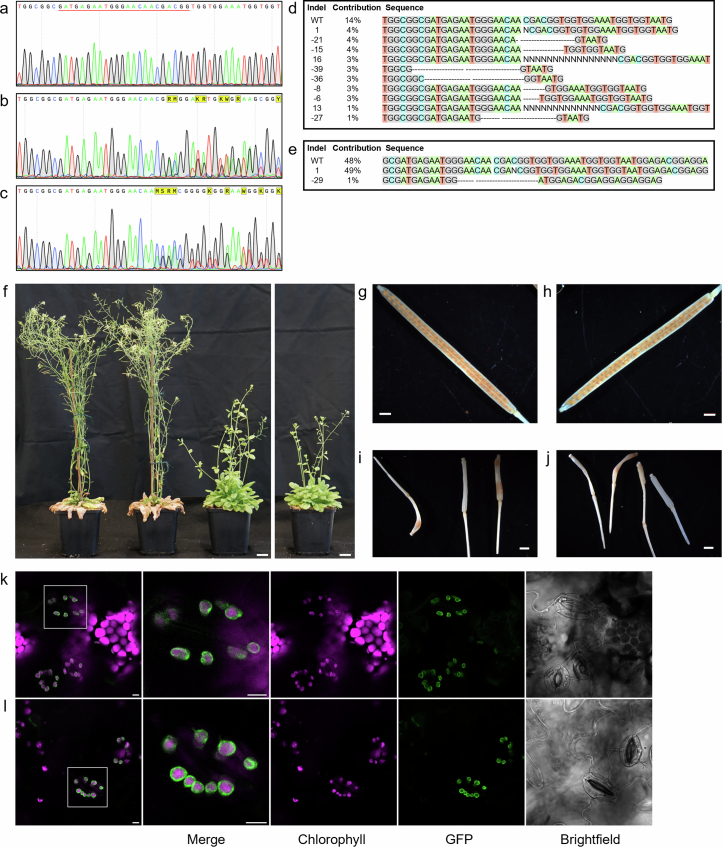
Verification of CRISPR/Cas9 dependent editing of *RER1.* Verification of CRISPR/Cas9 dependent editing event in the *RER1* sequence of (**a**) Col-0, (**b**) *re-6 x rer1* (-/+) plant 22 and (**c**) *re-6 x rer1* (-/+) plant 23. Guide sequence is underlined in red. Inference of CRISPR Edits (ICE) analysis of (**d**) *re-6 x rer1* (-/+) plant 22 and (**e**) *re-6 x rer1* (-/+) plant 23^62^. (**f**) Growth phenotype of adult Col-0, *re-6*, and *re-6 x rer1* (-/+) plant 22 and 23 (from left to right). Scale bar = 2 cm. Representative pictures of mature siliques of (**g**) Col-0, (**h**) *re-6*, (**i**) *re-6 x rer1* (-/+) plant 22, and (**j**) *re-6 x rer1* (-/+) plant 23. Scale bar = 1 mm. Mature siliques were harvested from three independent Col-0 and *re-6* plants, and one *re-6 x rer1* (-/+) plant each. Localization of (**k**) RE1 and (**l**) RER1-GFP fusion constructs (green) with chlorophyll autofluorescence (magenta) stably expressed in *Arabidopsis*. Whole seedlings were imaged 15 days after germination Scale bar = 5 µm. The experiment was independently repeated at least three times, with consistent results^63–70^.

Mutants deficient in *RE1* (*re-6*), but not in *RER1* (*rer1-1*, *rer1-2* and *rer1-3*), exhibit a reticulate leaf phenotype, which is characterized by the presence of dark-green veins on a paler leaf lamina, due to a defect in leaf development^23,25,26^ (Fig. 2a,b). The reticulate leaf phenotype can be rescued by overexpressing *RE1* and, to a similar extent, *RER1*, in the *re-6* mutant background (Extended Data Fig. 2b–e and Supplementary Text). Expression analyses from our and other studies indicate that, although the *re-6* mutant phenotype appears to be leaf specific, the expression of *RE1* and *RER1* is not restricted to a single plant organ or developmental stage, suggesting that RE1 and RER1 have functions throughout the plant life cycle^23,25,27^ (Extended Data Fig. 2f–h and Supplementary Text). A homozygous double-knockout mutant of both the *RE1* and *RER1* genes is lethal. Hemizygous mutants with a heterozygous mutation in *RER1* generated by CRISPR–Cas9 in the homozygous *re-6* mutant background exhibit the reticulate leaf phenotype and are further developmentally impaired in growth and seed production, as compared with the *re-6* mutant (Fig. 2b–d and Extended Data Fig. 3a–f). These mutants produced significantly shorter siliques with a significantly reduced seed set per silique compared with Col-0 and *re-6* (Fig. 2c,d and Extended Data Fig. 3g–j). Seeds obtained from these mutants were either wild type for *RER1* or showed a heterozygous mutation. Seeds carrying a homozygous mutation could not be isolated, indicating that a homozygous mutation in both genes is lethal, presumably during the stage of seed production.

Based on evidence from proteomic analyses and transit peptide predictions^28,29^, RE proteins probably localize to plastids. To independently validate the localization predictions of RE1 and RER1, we generated green fluorescent protein (GFP) fusions of each protein for transient expression in *Nicotiana benthamiana* (tobacco) leaves. GFP-tagged fusion proteins (depicted in green) formed a ring-like fluorescence pattern surrounding the chlorophyll autofluorescence signal (depicted in magenta) in isolated tobacco protoplasts (Fig. 2e,f). The same result was obtained when imaging *Arabidopsis* seedlings stably expressing the RE1 or RER1 GFP fusion proteins (Extended Data Fig. 3k,l). This is consistent with the inner envelope localization indicated by proteomics (Supplementary Text and Supplementary Fig. 1) and confirms the localization of both proteins to the plastid inner envelope.

### *re-6* has lower basic amino acid levels

**Fig. 3:**
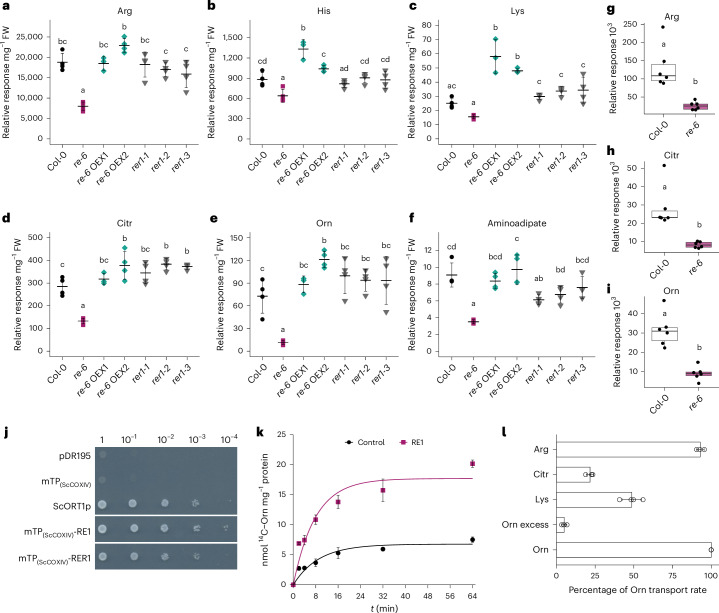
Metabolic characterization of *RE1*- and *RER1*-knockout mutants and *RE1* OEX lines, and in vitro transport characteristics of RE1. **a**–**f**, Relative metabolite levels per milligram of fresh weight (FW) of Arg (**a**), His (**b**), Lys (**c**), Citr (**d**), Orn (**e**) and aminoadipate (**f**) from 14-day old seedlings. Samples were harvested in the middle of the light period. Data are shown as mean ± s.d. of four biological replicates. Different letters indicate statistically significant differences between means (*P* < 0.05; one-way ANOVA with Tukey’s test). **g**–**i**, Relative responses of Arg (**g**), Citr (**h**) and Orn (**i**) in isolated chloroplasts from 3-week-old Col-0 and *re-6* plants. The relative response was normalized for chlorophyll content in μg ml^−1^. Data are presented as box and whiskers (minimum to maximum) of six biological replicates. Different letters indicate statistically significant differences between means (*P* < 0.05; one-way ANOVA with Tukey’s test). The box plots (**g**–**i**) show the median (horizontal line), interquartile range (box) and whiskers extending to 1.5× the interquartile range. Exact *P* values (**a**–**i**) are shown in the Source data with 95% confidence intervals. **j**, Growth behaviour of the yeast strain *arg11* transformed with pDR195 expressing the mitochondrial targeting peptide from the *S. cerevisiae* cytochrome c oxidase 4 (ScCOXIV), the *S. cerevisiae* mitochondrial Orn carrier (ScORT1p) and the mature forms of RE1 and RER1 (without predicted transit peptide) fused to the mitochondrial targeting peptide of ScCOXIV. Cells from positive transformants were inoculated to an initial OD_600_ of 1, diluted tenfold four times and grown on selective plates for 2 days at 30 °C. The experiment was independently repeated at least three times, with consistent results. **k**, Representative Orn uptake of RE1. Proteoliposomes were preloaded with 20 mM Orn. Data are shown as mean ± s.d. of technical triplicates. **l**, Substrate specificity of RE1. Proteoliposomes were preloaded with 20 mM Orn, Arg, Citr or Lys. Transport was measured against [^14^C]-Orn. The Orn transport rate was set to 100%. As a control, Orn was added to the transport mix in excess (Orn excess). Data are shown as mean ± s.d. of biological triplicates. Source data

**Extended Data Fig. 4:**
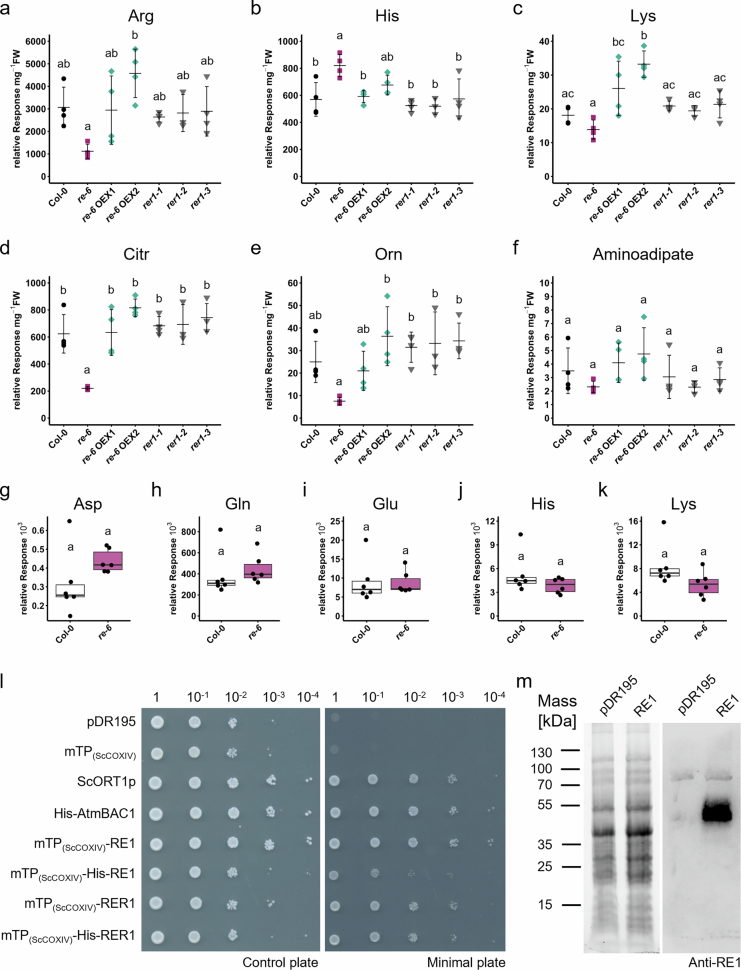
Metabolic characterization in rosette leaves and functional complementation of RE1 and RER1 in the *arg11* yeast mutant. Relative metabolite levels per mg fresh weight (FW) of (**a**) Arg, (**b**) His, (**c**) Lys, (**d**) Citr, (**e**) Orn, and (**f**) Aminoadipate from 4-week-old rosette leaves of Col-0, *re-6*, *rer1-1*, *rer1-2*, *rer1-3*, *re-6* OEX1 and *re-6* OEX2. Samples were harvested in the middle of the light period. Data are shown as mean ± SD of four biological replicates. Different letters indicate statistically significant differences between means (*P* < 0.05; one-way ANOVA with Tukey’s test). Relative response of (**g**) Asp, (**h**) Gln, (**i**) Glu, (**j**) His, and (**k**) Lys in isolated chloroplasts from 3-week-old Col-0 and *re-6* plants. Relative response was normalized for chlorophyll content in μg/mL. Data are presented as box and whiskers (min to max) of six biological replicates. Different letters indicate statistically significant differences between means (*P* < 0.05; one-way ANOVA with Tukey’s test). The boxplots (**g**–**k**) show the median (horizontal line), interquartile range (box), and whiskers extending to 1.5× the IQR. Exact *P* values (**a**–**k**) are shown in source data with 95% confidence intervals. (**l**) Growth behavior of the yeast strain *arg11* transformed with pDR195 expressing the mitochondrial targeting peptide from the *S. cerevisiae* cytochrome c oxidase 4 (ScCOXIV), the *S. cerevisiae* mitochondrial Orn carrier (ScORT1p), the mitochondrial basic amino acid carrier from *Arabidopsis* (AtmBAC1) fused with an N-terminal His-tag, and the mature forms of RE1 and RER1 fused to the mitochondrial targeting peptide of ScCOXIV with and without a N-terminal His-tag. Cells from positive transformants were inoculated to an initial OD_600_ of 1, diluted tenfold four times, and grown on control and selective plates for 2 d at 30 °C. (**m**) Verification of protein expression. Mitochondria were isolated from the yeast strain *arg11* transformed with pDR195 and pDR195 expressing the mature form of RE1 fused to the mitochondrial targeting peptide of ScCOXIV. 25 µg mitochondrial protein was separated by SDS-PAGE and transferred onto a nitrocellulose membrane. RE1 protein was visualized by immunodetection with an antibody raised against the RE1 protein. Source data

To investigate the physiological consequences of *RE1* and *RER1*deficiency in *Arabidopsis*, we analysed the amino acid contents in 14-day-old seedlings, and 4-week-old rosette leaves of Col-0, *re-6*, *re-6* OEX1, *re-6* OEX2, *rer1-1*, *rer1-2* and *rer1-3*. Amino acid levels were significantly altered in the *re-6* mutant compared with the wild type. Basic amino acids, Arg, His, Lys and the non-proteinogenic amino acids Citr and Orn, were significantly reduced in *re-6* mutant seedlings compared with the wild type (Fig. 3a–e and Supplementary Table 4). In mature rosette leaves, the Citr content was significantly reduced in *re-6*, whereas the levels of Arg, Lys and Orn remained unchanged (Extended Data Fig. 4a,c–e and Supplementary Table 5). Notably, the His content was significantly increased in *re-6* leaves compared with the wild type (Extended Data Fig. 4b). Although the basic amino acid content was altered in the *re-6* mutant, the levels of their precursors Asp, Glu and Gln remained unaffected (Supplementary Table 4). Interestingly, we observed a significant reduction in aminoadipate levels in the *re-6* mutant seedlings but not mature leaves (Fig. 3f and Extended Data Fig. 4f), suggesting a link to reduced Lys availability, as aminoadipate is a product of Lys degradation^30^. The metabolic defects of *re-6* were completely rescued by overexpressing *RE1* in the *re-6* mutant background (Fig. 3a–f, Extended Data Fig. 4a–f and Supplementary Tables 4 and 5). Moreover, *RE1* overexpression led to a significant increase of Citr, His, Lys, Orn, Phe and Trp levels (Supplementary Table 4), indicating a complex interaction of amino acid metabolism in vivo. The amino acid content of the *rer1* mutants was not affected (Fig. 3a–f, Extended Data Fig. 4a–f and Supplementary Tables 4 and 5).

We further analysed metabolite levels in chloroplasts isolated from 3-week-old Col-0 and *re-6* plants. The contents of Arg, Citr and Orn were significantly reduced in isolated chloroplasts from the *re-6* mutant compared with the wild type (Fig. 3g–i), indicating an overall reduced availability of basic amino acids in the *re-6* mutant. The levels of other amino acids such as Asp, Gln, Glu, His and Lys remained unaffected (Extended Data Fig. 4g–k and Supplementary Table 6). Proteomics showed that Arg and Lys de novo biosynthetic enzymes were differentially abundant in isolated chloroplasts of the *re-6* mutant compared with the wild type^31^. Together with the co-expression pattern of *RE1* (Supplementary Table 3) and the reduced amounts of basic amino acids in *re-6* seedlings and isolated chloroplasts compared with wild-type levels (Fig. 3a–i), this prompts the hypothesis that RE1 functions as plastidial basic amino acid carrier in *Arabidopsis*.

### RE1 transports basic amino acids in vitro

We next tested the hypothesis that RE1 functions as a basic amino acid carrier. To this end, we used the *Saccharomyces cerevisiae* mutant *arg11*, which lacks the mitochondrial Orn transporter ORT1p and displays Arg auxotrophy due to impaired Orn export from mitochondria to the cytosol^32,33^. To assess whether RE1 can complement this deficiency, we fused it to the mitochondrial transit peptide of ScCOXIV and expressed the construct under the control of the *PMA1* promoter in *arg11*. RE1 complemented the growth deficiency of *arg11* to the same extent as ORT1p, the native yeast carrier, while neither the empty vector nor the ScCOXIV peptide alone restored growth (Fig. 3j, Extended Data Fig. 4l and Supplementary Text).

**Extended Data Fig. 5:**
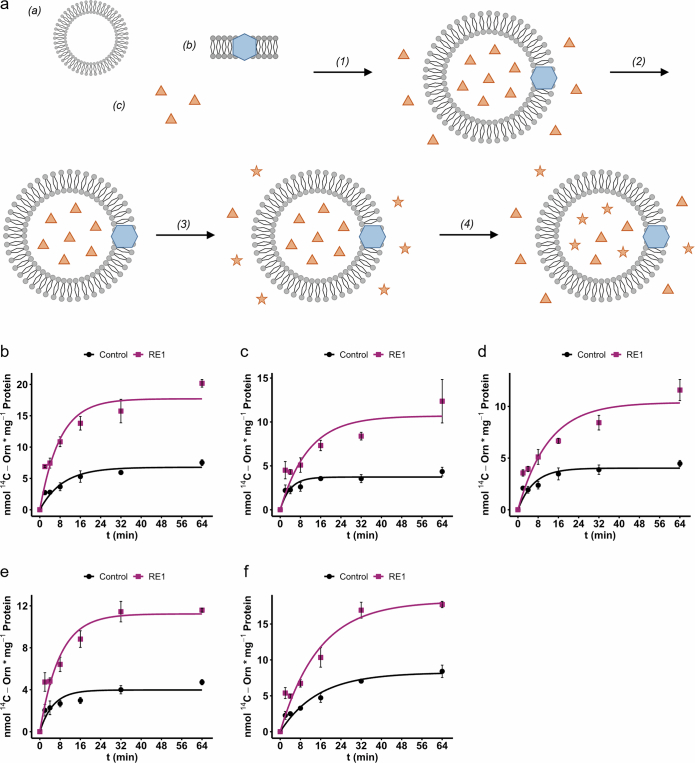
Transport analysis of RE1. (**a**) Representative scheme of the transport assay. Isolated mitochondrial membranes *(b)* were reconstituted *(1)* into liposomes *(a)* with or without 20 mM substrate *(c)*. External substrate was removed via size-exclusion chromatography *(2)*, and the transport reaction was initiated by diluting the proteoliposomes in transport mix *(3)* containing 0.2 mM substrate (orange triangle) and radioactive labeled substrate (orange star). Samples were taken *(4)* after 2, 4, 8, 16, 32 and 64 min and the radioactivity in the proteoliposomes was determined by liquid scintillation counting. (**b**–**f**) Orn uptake of RE1 of five independent biological replicates. Proteoliposomes were preloaded with 20 mM Orn. Data are shown as mean ± SD of technical triplicates. Source data

To further determine the mechanism by which RE1 restores the Orn uptake deficiency of the *arg11* yeast mutant, we isolated mitochondria from the *arg11* yeast mutant expressing the RE1 protein and reconstituted the isolated mitochondrial membranes into liposomes (Extended Data Figs. 4m and 5a). These RE1-containing liposomes exchanged Orn for radiolabelled Orn in independent biological replicates, indicating that expression of RE1 restores mitochondrial Orn uptake in the *arg11* yeast mutant (Fig. 3k, Extended Data Fig. 5b–f and Supplementary Text). Furthermore, reconstituted mitochondrial membranes containing the RE1 protein were able to exchange Arg, Citr and Lys against radiolabelled Orn (Fig. 3l). These results establish RE1 as the first identified plastidial basic amino acid carrier in *Arabidopsis*. The closest homologue of RE1, RER1, complemented *arg11* in a similar manner (Fig. 3j, Extended Data Fig. 4l and Supplementary Text), suggesting a possible functional similarity.

### *re-6* is hypersensitive towards exogenous basic amino acids

**Fig. 4:**
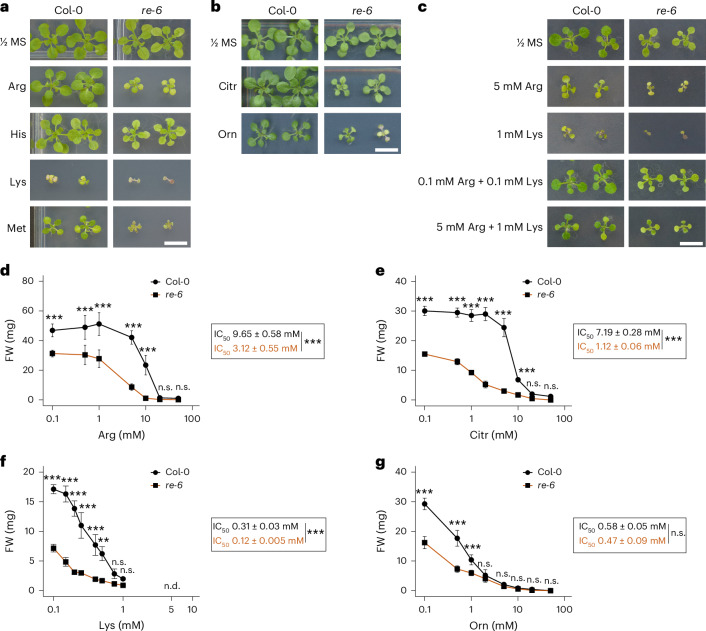
Phenotype of wild-type and *re-6*-knockout mutant in plants supplemented with exogenous amino acids. **a**,**b**, Growth-sensitive phenotype of Col-0 and *re-6* on ½ MS plates supplemented with 1 mM exogenous Arg, His, Lys and Met (**a**) and Citr and Orn (**b**). Pictures were taken after 3 weeks of growth. **c**, Growth analysis of Col-0 and *re-6* on plates supplemented with a combination of Arg and Lys. Pictures were taken after 2 weeks of growth. Scale bar, 1 cm. The experiments were independently repeated at least three times, with consistent results. **d**–**g**, Growth analysis of Col-0 and *re-6* seedlings on plates supplemented with different concentrations of Arg (**d**), Citr (**e**), Lys (**f**) and Orn (**g**). Fresh weight (FW) was measured after 3 weeks of growth. Data are shown as mean ± s.d. Asterisks indicate statistically significant differences between genotypes at the same concentration (***P < 0.001; **P < 0.01; n.s., not significant; n.d., not detected; two-way ANOVA with Šidák’s test). IC_50_ values were fitted using R (ref. ^59^). Asterisks indicate statistically significant differences (***P < 0.001; n.s., not significant; two-sided Student’s *t*-test). Exact *n* and *P* values are shown in the Source data with 95% confidence intervals. Source data

**Extended Data Fig. 6:**
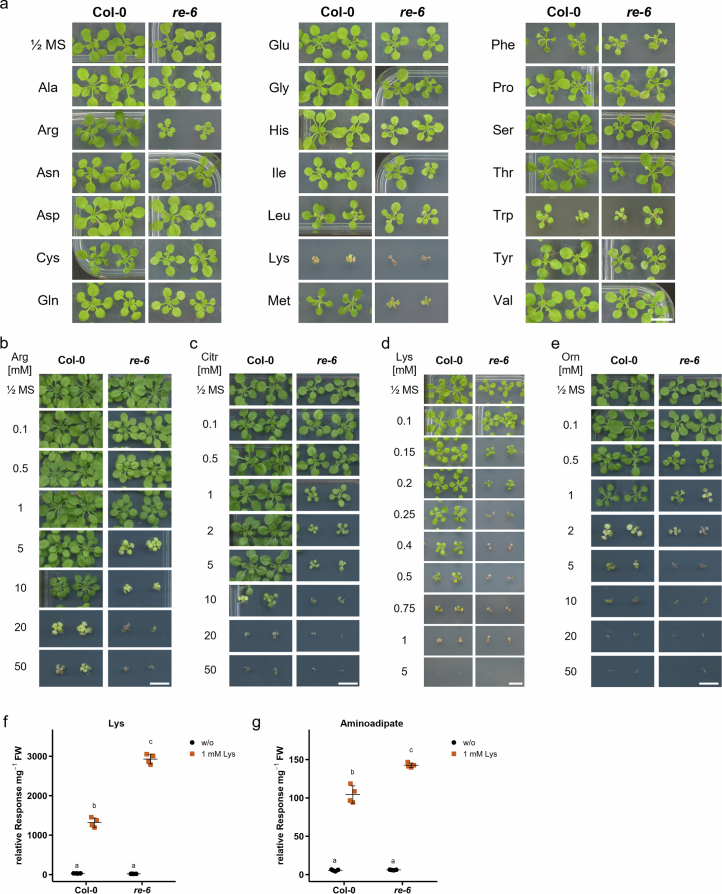
Amino acid feeding phenotype of the *RE1* mutant and metabolic changes upon Lys feeding. (**a**) Representative pictures of 3-week-old Col-0 and *re-6* plants grown on ½ MS supplemented with 1 mM proteinogenic amino acids. (**b**) Representative pictures of 4-week-old Col-0 and *re-6* plants grown on ½ MS supplemented with different Arg concentrations. Representative pictures of 3-week-old Col-0 and *re-6* plants grown on ½ MS supplemented with different (**c**) Citr, (**d**) Lys, and (**e**) Orn concentrations. Scale bar = 1 cm. The experiments were independently repeated at least three times, with consistent results. Relative (**f**) Lys and (**g**) aminoadipate level per mg fresh weight (FW) of 14-d old seedling with and without 1 mM Lys treatment for 2 d. Samples were harvested in the middle of the light period. Data are shown as mean ± SD of four biological replicates. Different letters indicate statistically significant differences between means (*P* < 0.05; two-way ANOVA). Exact *P* values (**f**, **g**) are shown in source data with 95% confidence intervals. Source data

Several *Arabidopsis* mutants with a reticulate leaf phenotype have been linked to defects in primary metabolism, particularly amino acid and nucleotide biosynthesis, and their phenotypes are often rescued by feeding pathway metabolites^34–37^. Based on this, we tested whether the *re-6*-knockout mutant phenotype could be rescued by supplementation with 1 mM each of all proteinogenic amino acids, as well as the non-proteinogenic amino acids Orn and Citr. Contrary to our expectations, the reticulate leaf phenotype of the *re-6* mutant could not be rescued under any of these conditions nor when a combination of Arg and Lys was applied (Fig. 4a–c and Extended Data Fig. 6a). Instead, feeding basic amino acids or Met impaired growth of the *re-6* mutant (Fig. 4a,b), with a significant, dose-dependent reduction in growth on plates supplemented with Arg, Citr, Lys and Orn, reflected in lower IC_50_ values in *re-6* compared with wild type (Fig. 4d–g and Extended Data Fig. 6b–e). The negative effect was most pronounced when exogenous Lys was supplied at concentrations ranging between 0.15 and 0.4 mM. At 0.4 mM, *re-6* mutant seedlings germinated but remained arrested at the cotyledon state, without progressing to photoautotrophic growth (Extended Data Fig. 6d). To exclude impaired Lys degradation as a cause, we conducted a short-term Lys feeding experiment in liquid medium and quantified Lys catabolites by liquid chromatography–tandem mass spectrometry. After 2 days of Lys feeding, both wild type and *re-6* showed a significant accumulation of aminoadipate, a Lys catabolite, indicating that *re-6* can efficiently degrade Lys (Extended Data Fig. 6f,g).

**Extended Data Fig. 7:**
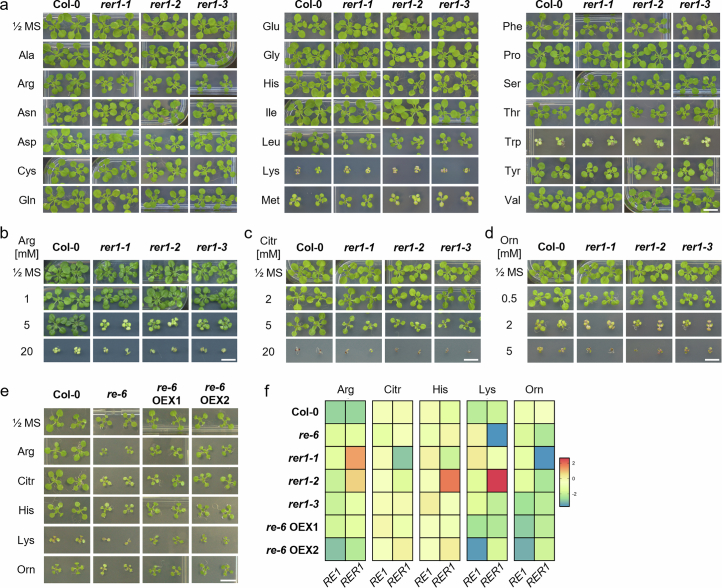
Amino acid feeding phenotype of *RER1* mutants and *RE1* OEX lines. (**a**) Representative pictures of 3-week-old Col-0 and *RER1* mutant plants grown on ½ MS supplemented with 1 mM proteinogenic amino acids. Representative pictures of 3-week-old Col-0 and *RER1* mutant plants grown on ½ MS supplemented with different (**b**) Arg, (**c**) Citr, and (**d**) Orn concentrations. Scale bar = 1 cm. (**e**) Representative pictures of 2-week-old Col-0, *re-6*, *re-6* OEX1 and *re-6* OEX2 on ½ MS plates supplemented with 1 mM exogenous Arg, Citr, His, Lys, and Orn. Scale bar = 1 cm. The experiments were independently repeated at least three times, with consistent results. (**f**) Relative *RE1* transcript levels in 14-d-old Col-0, *re-6* OEX1 and *re-6* OEX2 seedlings grown with or without the supplementation of 1 mM Arg, Citr, His, Lys, or Orn. Data are shown as log_2_ fold change of stressed versus control conditions. Source data

*rer1* mutants showed significantly reduced growth on plates supplemented with exogenous Arg and Citr, albeit at higher concentrations than *re-6* (Extended Data Fig. 7a–d), consistent with the absence of the reticulate leaf and metabolic phenotype in *rer1*. As their phenotype was less pronounced, *rer1* lines were not included in subsequent experiments. Overexpression of *RE1* in *re-6* fully restored the growth phenotype (Extended Data Fig. 7e). We further analysed *RE1* and *RER1* mRNA amounts in the different mutant lines grown on plates supplemented with Arg, Citr, His, Lys or Orn. Expression analysis revealed that *RE1* and *RER1* mRNA levels were generally lower in seedlings grown on plates supplemented with basic amino acid compared with control conditions (Extended Data Fig. 7f).

### Lys and Citr impact amino acid biosynthesis in *re-6*

**Fig. 5:**
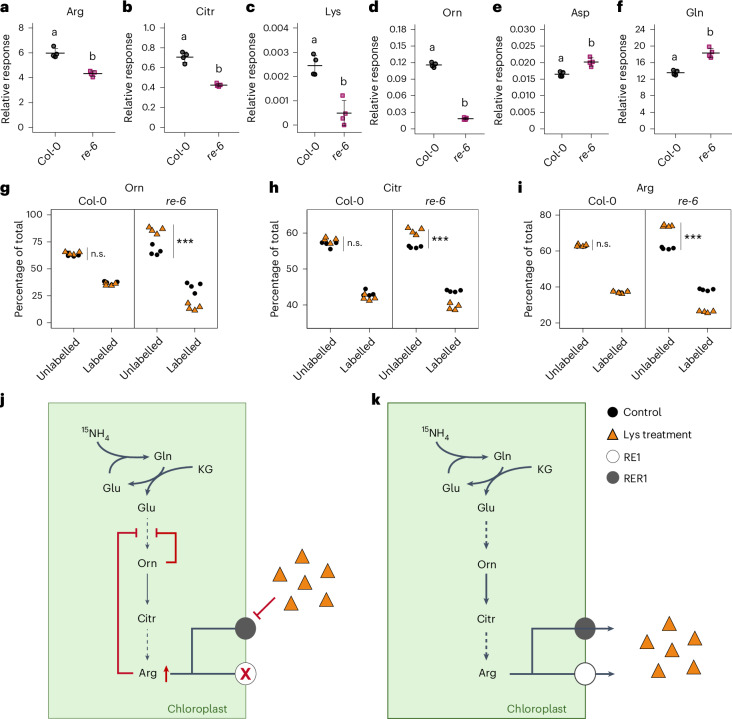
De novo basic amino acid biosynthesis is altered in the *re-6* mutant after Lys treatment. **a**–**f**, Relative responses of the absolute amounts of labelled Arg (**a**), Citr (**b**), Lys (**c**), Orn (**d**), Asp (**e**) and Gln (**f**) in 14-day-old Col-0 and *re-6* seedlings 2 days after labelling with 4 mM ^15^NH_4_Cl. Data are shown as mean ± s.d. Different letters indicate statistically significant differences between means (*P* < 0.05; one-way ANOVA with Tukey’s test). **g**–**i**, The percentage of unlabelled and labelled Orn (**g**), Citr (**h**) and Arg (**i**). Metabolites were extracted from the green tissue of 14-day-old seedlings, 2 days after labelling with 4 mM ^15^NH_4_Cl with (orange triangles) or without (black circles) 1 mM Lys. Asterisks indicate statistically significant differences (n.s., not significant; ***P < 0.001; two-sided Student’s *t*-test). Exact *P* values (**a**–**i**) are shown in the Source data with 95% confidence intervals. **j**,**k**, Schematic depictions of the impact of Lys treatment on de novo Arg, Citr and Orn biosynthesis in the *re-6* mutant (**j**) and the wild type (**k**). Reduced biosynthesis of Arg, Citr and Orn in the *re-6* mutant due to a feedback inhibition in their biosynthetic pathway (red line) caused by an accumulation of Arg (red arrow) as a result of a knockout of *RE1* (white circle with red cross) and an inhibition of RER1 (grey circle) transport activity by excess Lys (orange triangles) is emphasized by a reduced line thickness. KG, α-Ketoglutarate. Source data

**Extended Data Fig. 8:**
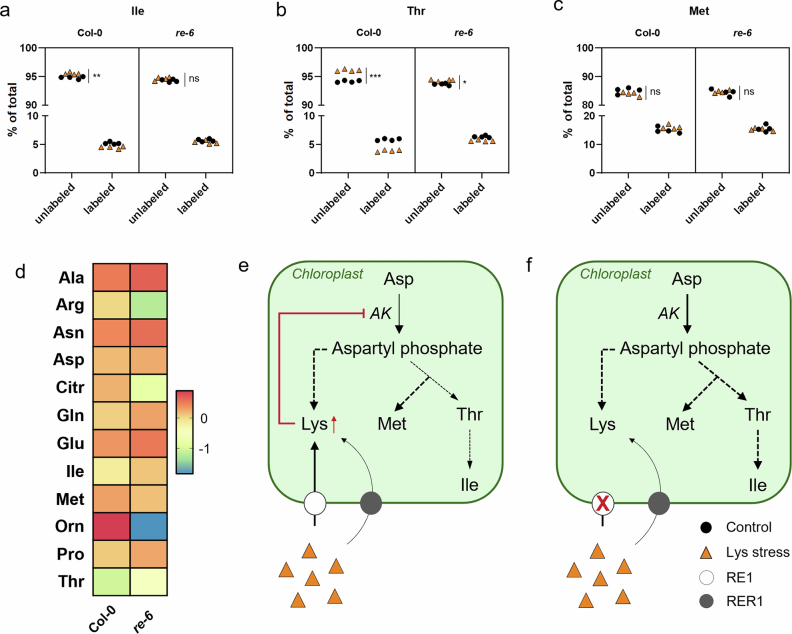
*De novo* amino acid biosynthesis changes in the wildtype and the *re-6* mutant after Lys treatment. Percent of unlabeled and labeled (**a**) Ile, (**b**) Thr, and (**c**) Met. Metabolites were extracted from the green tissue of 14-day old seedlings, two days after labeling with 4 mM ^15^NH_4_Cl with (orange triangles) or without (black circles) 1 mM Lys. Data are shown as four biological replicates. Asterisks indicate statistically significant differences (ns, not significant; *P < 0.05, **P < 0.01, ***P < 0.001; two-sided Student’s *t*-test). Exact *P* values (**a**–**c**) are shown in source data with 95% confidence intervals. (**d**) Metabolic changes of Col-0 and *re-6* after Lys treatment. Data are shown as log_2_ fold change of Lys treatment versus control conditions. Schematic depiction of the impact of Lys treatment on *de novo* Thr, Ile, and Met biosynthesis in the (**e**) wildtype and (**f**) the *re-6* mutant. The reduced biosynthesis of Thr and Ile in the wildtype due to a feedback inhibition in their biosynthetic pathway (red line) caused by an accumulation of Lys (red arrow) in the plastids by excess Lys treatment (orange triangles) is emphasized by a reduced line thickness. In the *re-6* mutant, Lys cannot be efficiently transported into the plastids due to a knockout of *RE1* (white circle with red cross). Source data

Despite the reduced content of basic amino acids in the *re-6* mutant (Fig. 3a–i), exogenous application of these amino acids further exacerbated the phenotype (Fig. 4a,b), suggesting that their limited availability is not the sole cause of the reticulate leaf phenotype. To further investigate the effect of the *re-6* mutation on amino acid biosynthesis and to understand the metabolic impact of excess Lys on amino acid biosynthesis in the *re-6* mutant, we performed a ^15^N-ammonium chloride labelling experiment and assessed the incorporation of the label into de novo synthesized amino acids. To this end, wild-type and *re-6* seedlings were incubated in liquid medium with or without 4 mM ^15^NH_4_Cl and/or 1 mM Lys. Under control conditions, the absolute amounts of labelled Arg, Citr, Lys and Orn were significantly lower in the *re-6* mutant compared with wild type (Fig. 5a–d), indicating a decreased biosynthesis rate in *re-6* (Supplementary Table 7). By contrast, levels of labelled Asp and Gln, precursors of Lys and Arg, were significantly increased in the *re-6* mutant, suggesting an upregulation of their biosynthesis (Fig. 5e,f and Supplementary Table 7). Notably, after Lys treatment, the relative fraction of ^15^N-labelled Arg, Citr and Orn was significantly reduced in the *re-6* mutant but remained unchanged in the wild type (Fig. 5g–i). Conversely, the relative fraction of ^15^N-labelled Ile was significantly reduced in the wild type but not the *re-6* mutant, while ^15^N-labelled Thr was reduced in both genotypes, with a more pronounced decrease in the wild type under those conditions (Extended Data Fig. 8a,b). Lys treatment had no effect on the de novo biosynthesis of Ala, Asn, Asp, Glu, Gln, Met and Pro, in either genotype (Extended Data Fig. 8c and Supplementary Table 7). Overall, Lys treatment resulted in increasing amino acid levels with two exceptions: Thr, which decreased in both genotypes, and Arg, Citr and Orn, which decreased only in the *re-6* mutant (Extended Data Fig. 8d). The reduction in Ile and Thr content in the wild type was significantly higher than in the *re-6* mutant. This aligns with a reduction of the fraction of ^15^N-labelled Ile and Thr and a reduced biosynthesis of both amino acids in the wild type (Extended Data Fig. 8a,b). The reduction of Arg, Citr and Orn levels in the *re-6* mutant after Lys treatment coincides with a reduction of the fraction of ^15^N-labelled Arg, Citr and Orn levels and a reduced biosynthesis of these amino acids in the *re-6* mutant (Fig. 5a,b,d).

**Fig. 6:**
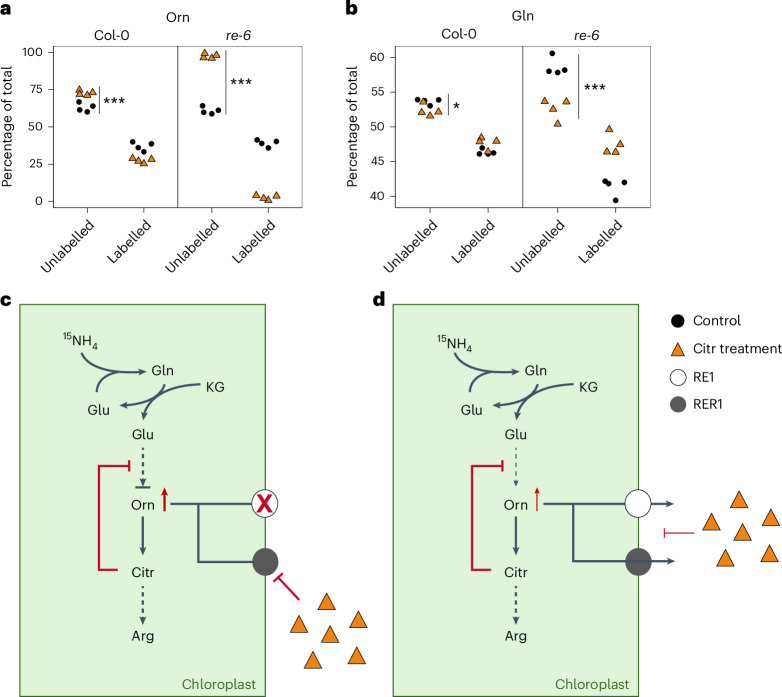
De novo amino acid biosynthesis is affected in the wild-type and the *re-6* mutant after Citr treatment. **a**,**b**, The percentage of unlabelled and labelled Orn (**a**) and Gln (**b**). Metabolites were extracted from the green tissue of 14-day-old seedlings, 2 days after labelling with 4 mM ^15^NH_4_Cl with (orange triangles) or without (black circles) 5 mM Citr. Data are shown as four biological replicates. Asterisks indicate statistically significant differences (*P < 0.05; ***P < 0.001; two-sided Student’s *t*-test). Exact *P* values (**a** and **b**) are shown in the Source data with 95% confidence intervals. **c**,**d**, Schematic depiction of the impact of Citr treatment on de novo biosynthesis in the *re-6* mutant (**c**) and the wild type (**d**). The slightly reduced biosynthesis of Orn in the wild type—due to feedback inhibition in its biosynthetic pathway (red line) caused by the accumulation of Orn (red arrow), which results from the partial inhibition of RE1 (white circle) and RER1 (grey circle) transport activity by excess Citr (orange triangles)—is indicated by a reduced line thickness. In the *re-6* mutant, Orn biosynthesis is almost completely blocked due to a feedback inhibition in its biosynthetic pathway (red line) caused by an accumulation of Orn (red arrow) as a result of a knockout of *RE1* (white circle with red cross) and an inhibition of RER1 (grey circle) transport activity by excess Citr (orange triangles). Source data

In an analogous approach, we analysed the effect of a 5 mM Citr treatment on de novo amino acid biosynthesis in wild-type and *re-6* seedlings. Similar to Lys treatment, absolute amounts of labelled Arg, Citr, Lys and Orn were significantly reduced in the *re-6* mutant compared with the wild type, confirming a reduced biosynthesis rate of these metabolites in *re-6* under non-stressed conditions (Supplementary Table 8). After Citr treatment, de novo biosynthesis of Orn was nearly abolished in the *re-6* mutant while it was only mildly affected in wild type (Fig. 6a). Citr treatment had a positive effect on de novo biosynthesis of Gln and His in the *re-6* mutant, increasing it by 6% and 5%, respectively (Fig. 6b and Supplementary Table 8). No effect of Citr treatment was observed on de novo biosynthesis of Ala, Asn, Asp, Glu, Ile, Leu, Lys, Met, Pro and Ser (Supplementary Table 8).

## Discussion

The *re* mutant, which was the first reticulate leaf mutant described in the literature^22^, has been widely used as a genetic marker owing to its distinctive phenotype. However, the function of the corresponding gene remained unresolved. Through a combination of yeast complementation analyses, functional protein reconstitution, and phenotypic and metabolic analyses of knockout mutants, we have identified RE1 and RER1 as plastidial basic amino acid carriers in *Arabidopsis*. Leaf reticulation in *Arabidopsis* has primarily been associated with mutations in genes involved in nucleotide and amino acid metabolism^26^. Interestingly, genes such as *RE1* and *RER1*, are mainly expressed within the vasculature and the surrounding cells, despite the reticulate phenotype being primarily visible in the mesophyll cells^23^. This highlights the importance of the vasculature in mesophyll cell and overall leaf development. It has been hypothesized that the vasculature provides and synthesizes primary metabolites, such as amino acids, to support mesophyll cell development^26,38,39^. As a result, the reticulate leaf phenotype can often be rescued by feeding such pathway metabolites^34–37^. However, in the *re-6* mutant, external supplementation of basic amino acids exacerbated the phenotype, suggesting a more complex metabolic disruption (Fig. 4a,b and Extended Data Fig. 6a). Interestingly, a double-knockout mutant of both genes, *RE1* and *RER1*, is lethal. Hemizygous knockout mutants are unable to produce siliques with a full seed set, suggesting that plastids provide amino acids for the synthesis of storage proteins. This is consistent with a significant reduction of the basic amino acid content in the *re-6* mutant seedling tissue (Fig. 3a–e), as a result of a reduced plastidial basic amino acid content (Fig. 3g–i) due to a reduced de novo biosynthesis rate of these amino acids (Fig. 5a–d and Supplementary Tables 7 and 8). This further demonstrates the essential role of plastid amino acid transport proteins in plant development and seed filling.

In addition, our steady-state metabolite profiling suggests that overexpression of *RE1* can lead to an increase in basic amino acid levels in seedlings (Fig. 3a–e). This is particularly interesting and might be a valuable addition to previous efforts increasing amino acid content in plants such as the engineering of feedback-insensitive enzymes^3^. Notably, we also observed an increase in non-basic amino acid levels (Supplementary Table 4), suggesting a more complex interaction of amino acid metabolism in vivo. Further in-depth metabolic characterization and phenotyping of the OEX lines will be essential to better understand these effects.

In plants, the biosynthesis of essential amino acids such as Lys is tightly regulated by feedback inhibition. Accumulation of Lys inhibits not only dihydrodipicolinate synthase, the committing step of the Lys biosynthesis pathway, but also Asp kinase, the initial enzyme in the branch responsible for aspartate-derived amino acids biosynthesis. As a result, de novo biosynthesis of Lys, Thr, Ile and Met is affected^5^. Our results corroborate the regulatory role of Lys in the wild type, as we observed a significant reduction in de novo Ile and Thr biosynthesis (Extended Data Fig. 8a,b). Based on our data, we propose two potential mechanisms by which excess Lys could inhibit their biosynthesis: (1) Lys import into the chloroplasts via RE1 and/or RER1 (Extended Data Figs. 8e) or (2) inhibition of Lys export by cytosolic Lys accumulation. Either mechanism could lead to intrachloroplastidic Lys accumulation and subsequent feedback inhibition of Asp kinase. While de novo biosynthesis of Met was not significantly affected under our conditions (Extended Data Fig. 8c), this may be due to its relatively low synthesis rates (Supplementary Table 7).

Interestingly, the *re-6* mutant displayed no significant reduction in de novo Ile biosynthesis after Lys treatment (Extended Data Fig. 8a), suggesting that Lys does not efficiently accumulate in the chloroplasts, probably due to the absence of RE1 (Extended Data Fig. 8f). However, we observed significant reductions in the de novo biosynthesis of Arg and its precursors Citr and Orn after Lys treatment in *re-6* but not the wild type (Fig. 5g–i). This observation is intriguing because no direct link is known to exist between de novo biosynthesis of Lys and Arg, Citr and Orn. While the mechanistic link between Lys and Arg metabolism remains unclear, one possible explanation is that impaired export of Arg and its precursors in the absence of RE1—combined with inhibition of RER1 after Lys treatment—leads to a feedback inhibition of Arg de novo biosynthesis^40^ (Fig. 5j). In the wild type, excess Lys is not sufficient to inhibit both RE1 and RER1. Therefore, de novo biosynthesis of Arg, Citr and Orn is not affected (Fig. 5k). Although the exact function of RER1 cannot be definitively established from our data, genetic evidence (Fig. 2b–d and Extended Data Fig. 2d,e) and yeast complementation assays (Fig. 3j and Extended Data Fig. 4l) support a similar role to RE1. We therefore propose that RER1 is functionally related to RE1 and may compensate for its loss under certain conditions.

**Extended Data Fig. 9:**
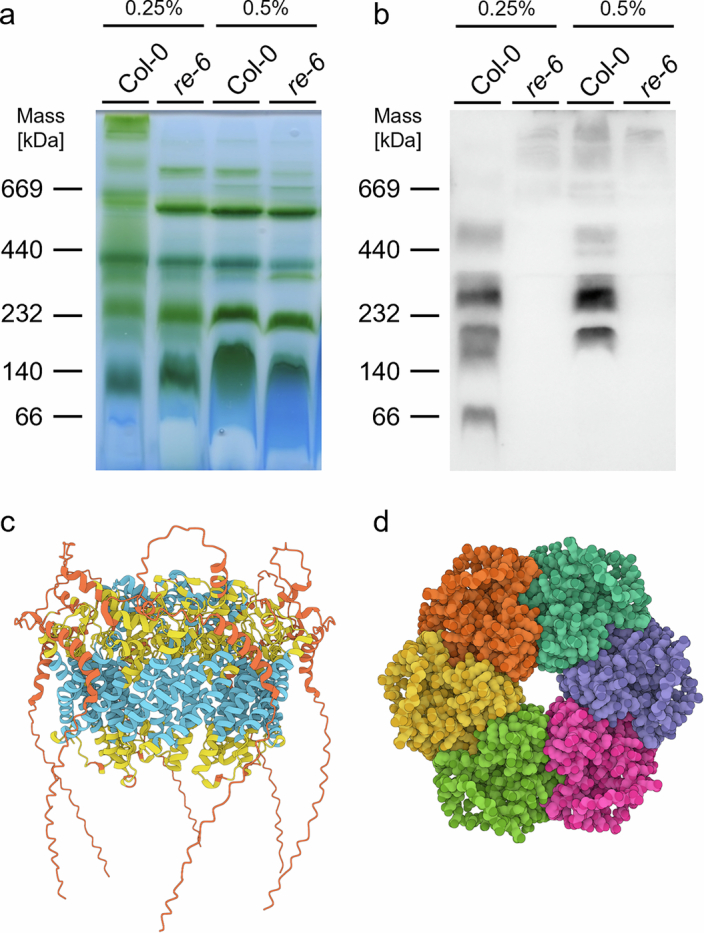
BN PAGE analysis of isolated chloroplasts and prediction of a homohexameric RE1 complex. (**a**) Fractionation of solubilized chloroplasts isolated from Col-0 and *re-6* rosette leaves on a BN-PAGE. Isolated chloroplasts equivalent to 10 µg chlorophyll were solubilized with DDM to a final concentration of 0.25% (w/v) or 0.5% (w/v), respectively, and separated by BN PAGE. (**b**) Immunological detection of the RE1 high molecular weight complex. Proteins separated by BN-PAGE were transferred onto a nitrocellulose membrane. RE1 protein was visualized by immunodetection with an antibody raised against the RE1 protein. The experiment was independently repeated three times, with consistent results. Structural prediction of a homohexameric RE1 complex using AlphaFold 3^24^. (**c**) Sideview of the complex. (**d**) Top view of the complex. Separate colors indicate RE1 subunits.

A similar pattern was observed after Citr treatment: de novo biosynthesis of Orn was diminished in both genotypes but was nearly abolished in *re-6* (Fig. 6a and Supplementary Table 8). This suggests that Orn cannot be efficiently exported from the plastids of the *re-6* mutant after Citr treatment, probably due to (1) the absence of RE1 and (2) an inhibitory effect of Citr on RER1 transport activity (Fig. 6c,d). These observations support a model where RE1 transports multiple basic amino acids in vivo, probably with distinct affinities. In addition, we propose that RER1 shares this role. This conclusion is supported by data from the yeast complementation analysis and the functional reconstitution in liposomes (Fig. 3j–l). The identification of a suitable expression system enabling correct assembly into the putative homohexameric complex (Extended Data Fig. 9) and the in-depth biochemical analyses of both proteins are the aim of future studies.

Among the nine proteinogenic amino acids synthesized exclusively de novo in the plastids, we identified transport proteins, RE1 and RER1, that facilitate the translocation of two of them, Arg and Lys, across the plastid inner envelope. RE and RER1 belong to a previously uncharacterized protein family defined by the DUF3411, which is found exclusively in plastid-containing organisms. This family is distinct from the three major protein families (the amino acid transporter family, the amino acid–polyamine–choline transporter family and the usually multiple acids move in and out transporter family) that comprise the majority of plant amino acid transport proteins identified so far. While the detailed transport mechanism remains unresolved, our results support the role of RE1 and RER1 as dedicated transporters for basic amino acids. We propose that the four additional members of the DUF3411 family, which presumably localize to the inner plastid envelope, may also function as amino acid transport proteins, but possess distinct substrate specificities. The structural and kinetic characterization of these proteins remains an important goal for future studies.

## Methods

### Plant growth conditions

*Arabidopsis* ecotype Col-0 and T-DNA insertion lines *re-6* (Salk_084529; http://signal.salk.edu/)^25^, *rer1-1* (Salk_126363; http://signal.salk.edu/)^23^, *rer1-2* (Salk_073984; http://signal.salk.edu/)^23^ and *rer1-3* (Salk_093173; http://signal.salk.edu/)^23^ were used in this study. Mutant seeds were obtained from the Nottingham *Arabidopsis* Stock Centre (NASC, https://*Arabidopsis*.info/). The T-DNA insertion lines were verified by PCR using gene-specific primer pairs and the left border primer of the T-DNA insertion (Supplementary Table 9).

*Arabidopsis* seeds were grown on half-strength Murashige and Skoog (0.5 MS) medium (pH 5.7) without sucrose supplemented with 0.8% (w/v) agar. Exogenous amino acids were added at a defined concentration where indicated. Seeds were surface-sterilized with sodium hypochlorite and subjected to cold stratification for 2 days at 4 °C. After germination, seedlings were grown for 10–21 days under a 12-h light/12-h dark photoperiod under 100 µmol m^−2^ s^−1^ light intensity, unless otherwise stated. Seedlings were transferred to soil after 14 days and grown under a 12-h light/12-h dark photoperiod under 130 µmol m^−2^ s^−1^ light intensity and 50% humidity if not stated otherwise. Samples were taken in the middle of the light period unless stated otherwise.

### Amino acid feeding and ^15^N-labelling experiments

Amino acid feeding experiments were performed on 0.5 MS medium (pH 5.7) supplemented with 0.8% (w/v) agar and exogenous amino acids at defined concentrations. Seedlings were grown for 14–28 days under a 12-h light/12-h dark photoperiod under 100 µmol m^−2^ s^−1^ light intensity. Samples for quantitative real-time PCR were taken in the middle of the light period.

For the ^15^NH_4_Cl-labelling experiment, *Arabidopsis* seedlings were transferred to liquid 0.5 MS medium (pH 5.7) with or without the addition of 1 mM Lys or 5 mM Citr, and/or 4 mM ^15^NH_4_Cl (Cambridge Isotope Laboratories) after 12 days and incubated for 2 days under a 12-h light/12-h dark photoperiod under 100 µmol m^−2^ s^−1^ light intensity. Seedlings were harvested in the middle of the light period, washed with ice-cold 0.9% (w/v) NaCl solution and rapidly frozen in liquid N_2_ for metabolite analysis.

### Generation of *RER1*-knockout mutants by CRISPR–Cas9

*RER1* (AT5G22790) knockout mutants were generated in the *re-6* mutant background using CRISPR–Cas9 technology. Four guides targeting exon 1 of *RER1* were selected using the CRISPR-P v2.0 web tool^41^. Sequences of the four selected guides are listed in Supplementary Table 9. All four guides were assembled as one transcript under the control of the U6-26 promoter using the polycistronic tRNA–gRNA (PTG) strategy^42^. The final vector contained the following expression cassettes: (1) phosphinothricin resistance, (2) Cas9 under the control of the egg-cell-specific EC1.2 promoter^43^, (3) the four guides under control of the U6-26 promoter in one construct using the PTG strategy^42^ and (4) a GFP under the control of the seed-specific At2S3 promoter^44^. All plasmids were generated by Golden Gate cloning using the MoClo tool kit^45^. The vectors containing the expression cassettes 1, 2 and 4, as well as the template for the PTG cassette were kindly provided by Claus Peter Witte (University of Hannover). The final vector was integrated into *Arabidopsis* *re-6* mutant via *Agrobacterium*-mediated transformation using the floral dip method^46^. Positive transformants were selected by seed GFP fluorescence and growth on 0.5 MS medium (pH 5.7) supplemented with 0.8% (w/v) agar and 7.5 µg ml^−1^ glufosinate-ammonium (Sigma-Aldrich). Mutant plants were identified by Sanger sequencing.

### Generation of *RE1* and *RER1* overexpression lines

*RE1-*overexpressing lines were generated in the *re-6* mutant background. The coding sequence of *AtRE1* (AT2G37860) was amplified from *Arabidopsis* cDNA using a proofreading polymerase and assembled into a plant expression vector with the *UBQUITIN10* promoter from *Arabidopsis*, and a C- or N-terminal GFP from *A. victoria*. *RER1*-overexpressing lines were generated in the *re-6* mutant background. The coding sequence of *AtRER1* (AT5G22790) was amplified from *Arabidopsis* cDNA using a proofreading polymerase and assembled into a plant expression vector with the *UBQUITIN10* promoter from *Arabidopsis* and N-terminal GFP from *A. victoria*. N-terminal GFP fusion was accomplished by integrating the GFP sequence between the predicted plastid transit peptide sequence and the mature sequence of *RE1* or *RER1*. The final constructs were integrated into *Arabidopsis* *re-6* mutant via *Agrobacterium*-mediated transformation using the floral dip method^46^. Positive transformants were selected by growth on 0.5 MS medium (pH 5.7) supplemented with 0.8% (w/v) agar and 50 µg ml^−1^ kanamycin.

### Generation and transient expression of GFP fusion constructs

Coding sequences of *AtRE1* and *AtRER1* were amplified from *Arabidopsis* cDNA using a proofreading polymerase and assembled with the *UBQUITIN10* promoter from *Arabidopsis*, a C-terminal GFP from *A. victoria* and the RbcS3C terminator from *S. lycopersicum* using Golden Gate cloning. Plasmids were generated with the MoClo tool kit^45^. *Agrobacterium tumefaciens* strain GV3103 transformed with the GFP fusion constructs were grown overnight at 30 °C in YEP medium (1% (w/v) yeast extract and 1% (w/v) peptone) at 200 rpm. Cells were collected by centrifugation (3,500*g*, 5 min) and resuspended to an optical density at 600 nm (OD_600_) of 0.5 in infiltration medium (10 mM 2-(*N*-morpholino)ethanesulfonic acid (MES)–KOH (pH 5.7), 10 mM MgCl_2_ and 200 µM acetosyringone). The abaxial side of 4–5-week-old tobacco leaves was infiltrated with the *Agrobacterium* suspension using a syringe. Two days after infiltration, protoplasts were isolated from infiltrated leaves. Leaves were cut into 0.5 × 0.5 cm pieces, vacuum infiltrated with digestion medium (20 mM MES–KOH (pH 5.7), 0.4 M mannitol, 20 mM KCl, 10 mM CaCl_2_, 1.5% (w/v) cellulase R-10 (Duchefa Biochemie), 0.4% (w/v) Macerozyme (Duchefa Biochemie) and 0.1% (w/v) bovine serum albumin) and incubated for 2 h at 30 °C. Isolated protoplasts were washed with wash buffer (4 mM MES–KOH (pH 5.7), 0.7 M mannitol and 15 mM MgCl_2_) and used for microscopy^47^. Microscopy images were acquired using Leica SP8 confocal microscope (Leica Microsystems). The following excitation/emission settings were used: GFP (488 nm/498–540 nm), chlorophyll A (488 nm/630–702 nm). Images were processed using Fiji^48^.

### Quantitative PCR

Total RNA from 14-day-old seedlings, 34-day-old rosette leaves, 14-day-old shoot and root tissue and 14-day-old seedlings grown on plates supplemented with 1 mM Arg, Citr, His, Lys or Orn was extracted using the RNeasy Plant Mini Kit (Qiagen). One microgram of total RNA was DNase treated with RNase-free DNase (Promega). One microgram of DNase-treated RNA was reverse-transcribed into cDNA using the LunaScript RT Super Mix Kit (New England Biolabs). Quantitative PCR was carried out using Luna Universal qPCR Master Mix (New England Biolabs) and a StepOnePlus Real-Time PCR thermocycle (Applied Biosystems). Gene-specific primers for *RE1*, *RER1* and the reference genes *PP2AA3* (AT1G13320)^49^ and *TIP41L* (AT4G34270)^49^ are listed in Supplementary Table 9. Mean normalized expression was calculated as described previously^50^.

### Chloroplast isolation, solubilization and BN-PAGE fractionation

Chloroplasts were isolated from approximately 4-week-old *Arabidopsis* Col-0 and *re-6* leaf tissue as described previously^31^. Chloroplasts equivalent to 10 µg of chlorophyll were pelleted by centrifugation for 10 min at 16,000*g* at 4 °C. The pellets were each resuspended in solubilization buffer B (50 mM imidazole-HCl (pH 7.0), 500 mM 6-aminocaproic acid and 1 mM EDTA) supplemented with *N*-dodecyl-β-d-maltoside (DDM) to a final concentration of 0.25% (w/v) or 0.5% (w/v) and incubated for 20 min at 4 °C. The samples were processed as described previously^31^. Fractionation of isolated solubilized chloroplasts was performed as described previously^31^. For protease protection assays, intact chloroplasts were incubated with 50 μg ml^−1^ thermolysin in the presence of 1 mM CaCl_2_ for 30 min or 25 μg ml^−1^ trypsin for 0–45 min at 4 °C. Reactions were terminated by adding 10 mM EDTA (thermolysin) or sodium dodecyl sulfate (SDS) loading buffer (trypsin). Samples were separated on a 12% SDS–polyacrylamide gel electrophoresis (PAGE). Proteins were transferred onto nitrocellulose membrane and analysed with primary anti-RE1 (1:2,000; Agrisera), primary anti-RbcL antibody (1:5,000, Agrisera) and secondary goat anti-rabbit-horse-radish peroxidase antibody (1:2,000; Merck Millipore) using standard protocols. Primary anti-RE1 antibody was raised against amino acids 73–86 at the N-terminal part of the RE1 protein.

### Chloroplast isolation for amino acid quantification

Chloroplasts for amino acid quantification were isolated from 3-week-old *Arabidopsis* leaves using the Minute Chloroplast Isolation Kit (Invent Biotechnologies) according to the manufacturer’s instruction. Chloroplasts were extracted from 150 mg tissue per replicate. The pellet after the second centrifugation step was frozen in liquid N_2_ and used for the quantification of amino acids.

### Generation of yeast complementation constructs

Coding sequences of the mature form of *AtRE1* (omitting the first 54 amino acids), of the mature form of *AtRER1* (omitting the first 29 amino acids) and of the mitochondrial basic amino acid carrier from *Arabidopsis* (AtmBAC1; AT2G33820) were amplified from *Arabidopsis* cDNA using a proofreading polymerase. Coding sequences of the mitochondrial ornithine carrier from *Saccharomyces cerevisiae* (ScORT1p; YOR130C), and the mitochondrial targeting peptide of the subunit 4 of the *Saccharomyces cerevisiae* cytochrome c oxidase (ScCOXIV; YGL187C; first 25 amino acids)^51^ was amplified from *Saccharomyces cerevisiae* gDNA using a proofreading polymerase. The PCR fragments of *AtRE1* and *AtRER1* with and without N-terminal 6xHis-tag were assembled with the PCR fragment of the mitochondrial targeting peptide of ScCOXIV into the yeast expression vector pDR195^52^ via Gibson cloning. The PCR fragments of *AtmBAC1* and *ScORT1p* were assembled with N-terminal 6xHis-tag into the yeast expression vector pDR195^52^ via Gibson cloning.

### Yeast complementation

The yeast strain Y02386/*arg11* (MATa; his3D1; leu2D0; met15D0; ura3D0; YOR130c::kanMX4; Euroscarf) carrying a deletion of the yeast gene *ARG11* encoding for a mitochondrial ornithine carrier^33^ was used for complementation in this study. The genotype of the mutant was verified by PCR using gene-specific primers and primers binding on the kanMX4 cassette (Supplementary Table 9). *arg11* was transformed with the complementation constructs. As negative controls, *arg11* was transformed with the empty vector pDR195 and the empty vector pDR195 expressing the mitochondrial targeting peptide of ScCOXIV. Positive yeast transformants were selected on uracil-free YNB medium (0.67% (w/v) yeast nitrogen base without amino acids) supplemented with 2% (w/v) glucose, 2% (w/v) BactoAgar and drop-out mix. Positive yeast transformants were validated by PCR using a vector-specific primer pair (Supplementary Table 9). Single colonies were inoculated in uracil-free YNB medium supplemented with 2% (w/v) glucose and drop-out mix and grown overnight at 30 °C with moderate shaking. Cells were inoculated in 50 ml uracil-free YNB medium supplemented with 2% (w/v) glucose and drop-out mix to an OD_600_ of 0.4. The cell suspension was incubated at 30 °C with moderate shaking (200 rpm) for 4 h. Cells were collected by centrifugation, washed three times with phosphate-buffered saline and adjusted to an OD_600_ of 1 using PBS. Cells were spotted on selection plates (0.17% (w/v) yeast nitrogen base without (NH_4_)_2_SO_4_, 5,000 mg l^−1^ (NH_4_)_2_SO_4_, 20 mg l^−1^ His, 100 mg l^−1^ Leu, 20 mg l^−1^ Met, 2% (w/v) glucose and 2% (w/v) BactoAgar) and incubated for 2–3 days at 30 °C.

### Yeast mitochondria isolation

Yeast mitochondria were purified from the *arg11* yeast mutant transformed with the empty vector control (pDR195) and the RE1 construct as described previously^53^. Yeasts were grown for 48 h in uracil-free YNB medium supplemented with 2% (v/v) glucose and drop-out mix. Purified mitochondria were resuspended in 1 ml SEM buffer (10 mM MOPS–KOH (pH 7.2), 250 mM sucrose and 1 mM EDTA), frozen in liquid nitrogen and stored at −80 °C.

### Reconstitution

Isolated yeast mitochondria were subjected to four freeze–thaw cycles. Mitochondrial membranes were pelleted by ultracentrifugation for 1 h at 100,000*g* at 4 °C. Membranes were resuspended in membrane resuspension buffer (50 mM HEPES–KOH (pH 7.5) and 5 mM MgCl_2_) and reconstituted into liposomes using the freeze–thaw procedure. In brief, 100 µg isolated yeast mitochondrial membrane protein were rapidly mixed with 3% (w/v) sonicated lipid (l-α-phosphatidylcholine from egg yolk, Sigma-Aldrich) in reconstitution buffer with (100 mM Tricine–KOH (pH 7.6), 30 mM K-gluconate and 20 mM substrate) or without (100 mM Tricine–KOH (pH 7.6) and 50 mM K-gluconate) preloading of substrate, frozen in liquid nitrogen and stored at −80 °C.

### Transport assays

Proteoliposomes were thawed at 24 °C, and external substrate was removed by size-exclusion chromatography using Sephadex G-75 columns (GE-Healthcare) preequilibrated with PD-10 buffer (10 mM Tricine–KOH, (pH 7.6), 100 mM Na-gluconate and 40 mM K-gluconate). Transport was initiated by adding ^14^C-labelled substrate and 0.2 mM non-labelled substrate to proteoliposomes with or without preloading of substrate. Uptake of external l-[^14^C]-ornithine (Hartmann Analytics) was measured at 30 °C. Transport was stopped by adding 140 µl of the mixture to preequilibrated size-exclusion columns. Liposomes were separated from external radioactivity by passing through Sephadex G-75 medium columns (Sigma-Aldrich) with PD-10 buffer. The radioactivity inside the liposomes was determined by liquid scintillation counting (Tri-Carb 4910 TR, PerkinElmer).

### SDS–PAGE and immunoblot analysis

Twenty-five micrograms of isolated yeast mitochondrial membranes or isolated chloroplasts were analysed by SDS–PAGE and immunoblot analysis with primary anti-RE1 (1:2,000; Agrisera), primary anti-RbcL (1:5,000, Agrisera) and secondary goat anti-rabbit-horse-radish peroxidase antibody (1:2,000; Merck Millipore) using standard protocols. Primary anti-RE1 antibody was raised against amino acids 73–86 at the N-terminal part of the RE1 protein. The antibody was used unpurified. To prevent aggregation of membrane proteins, isolated yeast mitochondrial membranes were incubated for 30 min at 30 °C in SDS-loading buffer before the analysis^54^.

### Amino acid measurements via HILIC–MS

Amino acid content was analysed from green tissue of 14-day-old seedlings and 4-week-old rosettes via hydrophilic interaction liquid chromatography coupled to mass spectrometry (HILIC–MS). Seedling material was ground to a fine powder using liquid N_2_. Metabolites were extracted from 30 mg of seedling material using a two-phase extraction protocol^55^. In brief, the frozen powder was quenched by adding 350 µl ice-cold extraction solution (CH_3_OH:CHCl_3_, 10:4.28) and incubated at −20 °C for 1 h with occasional mixing. Water-soluble components were extracted by adding 560 µl ice-cold ddH_2_O supplemented with 1.86 µM Phe-d_5_ and Val-d_8_ (Sigma-Aldrich). Samples were centrifuged for 4 min at 16,000*g* at 4 °C. The water-soluble phase was transferred into a new tube. The organic phase was reextracted with 560 µl ice-cold ddH_2_O. The combined water-soluble extracts were lyophilized, reconstituted in 500 µl deionized water and filtrated through a spin filter (pore size 0.2 µm). Samples were diluted 1:10 in injection solution (33% (v/v) acetonitrile and 66% (v/v) 10 mM ammonium formate (pH 3.0)). Amino acids were quantified using an Agilent (Santa Clara) 6490 Triple-Quadrupole mass spectrometer coupled to an Agilent 1260 bioinert HPLC system. The chromatographic separation was performed on a PEEK-coated SeQuant ZIC-HILIC column (Merck; 150 × 2.1 mm, 5 µm polymer) in combination with a ZIC-pHILIC guard column (Merck; 20 × 2.1 mm) at a constant flow rate of 0.2 ml min^−1^ with 200 mM ammonium formate (adjusted to pH 3.0 with formic acid) as stock solution diluted 1:10 in 80:10 water:acetonitrile as mobile phase A and diluted 1:10 in acetonitrile as mobile phase B. The injection volume was 5 µl. The autosampler was set to 8 °C and the column compartment to 45 °C. The mobile phase profile consisted of the following steps and linear gradients: 0.5 min 0% B, 0.5–17 min from 0% to 40% B, 17–20 min from 40% to 60% B, 20–23 min constant at 60% B. Within 3 min, the starting conditions of 0% B were reached. The equilibration time was set to 10 min. The mass spectrometer was used in positive mode with the following conditions: capillary voltage 2,000 V, nozzle voltage 500 V, 280 °C gas temperature at a flow of 17 l min^−1^, 400 °C sheath gas temperature at a flow of 12 l min^−1^, nebulizer pressure 25 psi. The detector electron multiplier voltage was set to (+)300 V.

### Targeted LC–HRMS analysis of amine-containing metabolites

The liquid chromatography high-resolution mass spectrometry (LC–HRMS)-based analysis of amine-containing compounds was performed using an acquity premier UHPLC chromatography system (Waters) coupled to a QE-Plus high-resolution mass spectrometer (Thermo Fisher Scientific). In brief, isolated chloroplasts were extracted by adding 350 µl of precooled (−20 °C) methanol and chloroform (7:3). The extracts were incubated for 1 h at −20 °C with occasional shaking before adding of 560 µl of ice-cold LC–MS water containing an internal amino acid standard at a final concentration of 5 µM. The mixture was vortexed and incubated on ice for 10 min before phase separation was induced by centrifugation at 21,000*g* for 10 min at 4 °C. The upper polar phase was transferred to a fresh 2-ml tube and completely dried in a vacuum concentrator, while the lower non-polar phase was used to determine the chlorophyll concentration^56^. The dried polar phase was solubilized in 100 µl LC–MS water, and 50 µl resolubilized polar phase was mixed with 25 µl of 100 mM sodium carbonate, followed by the addition of 25 µl 2% (v/v) benzoylchloride in acetonitrile, as reported previously^57^. The derivatized samples were thoroughly mixed and kept at a temperature of 20 °C until analysis.

For the LC–HRMS analysis, 2 µl of the derivatized sample was injected onto a 100 × 2.1 mm HSS T3 UPLC column (Waters). The flow rate was set to 400 µl min^−1^ using a binary buffer system consisting of buffer A (10 mM ammonium formate, 0.15% (v/v) formic acid in ultra low contaminants MS-grade water). Buffer B consisted of acetonitrile. The column temperature was set to 35 °C, while the LC gradient was as follows: 0% B at 0 min, 0–15% B 0–4.1 min; 15–17% B 4.1–4.5 min; 17–55% B 4.5–11 min; 55–70% B 11–11.5 min, 70–100% B 11.5–13 min; B 100% 13–14 min; 100–0% B 14–14.1 min; 0% B 14.1–19 min; 0% B. The mass spectrometer (Q-Exactive Plus) was operating in positive ionization mode recording the mass range *m*/*z* 100–1,000. The heated electrospray ionization source settings of the mass spectrometer were as follows: spray voltage 3.5 kV, capillary temperature 300 °C, sheath gas flow 60 AU, aux gas flow 20 AU at 330 °C and sweep gas 2 AU. The radiofrequency lens was set to a value of 60.

### Data analysis and statistical methods

The LC–MS data analysis was performed using the Skyline software (Skyline 24.1.0.199). The identity of each compound was validated by authentic reference compounds. For LC–HRMS data analysis, the area of the protonated [M + nBz + H]^+^ (nBz stands for the number of benzoyl moieties attached to each compound) of each required compound was extracted and integrated using a mass accuracy <5 ppm and a retention time tolerance of <0.1 min as compared with the independently measured reference compounds. Integrated peak areas of HILIC–MS and LC–HRMS amino acid analyses were normalized to internal standards and are shown as relative responses per milligram of fresh weight of the respective samples. For the ^15^NH_4_Cl-labelling experiment, the peak areas corresponding to the [M]^+^ (unlabelled) through [M + *N*]^+^ (labelled, where *N* is the number of labelled nitrogen atoms in the metabolite) were integrated, normalized to internal standards and corrected for the natural abundance using the R (4.3.1) package IsoCorrectoR^58^. Absolute amounts of a labelled metabolite as taken from the m1 pool are shown as relative responses. The total fraction of labelled and unlabelled molecules of a metabolite are expressed as percentages of the total.

Statistical significance was assessed as described in the figure legends using individual *Arabidopsis* plants or individual yeast expression events as biological replicates.

### Reporting summary

Further information on research design is available in the Nature Portfolio Reporting Summary linked to this article.

## Supplementary information

Supplementary Information Supplementary Text, Supplementary Fig. 1 and descriptions for Supplementary Tables 1–9.

Reporting Summary

Supplementary Tables 1–9 Supplementary Tables 1–9 in one workbook with separate tabs.

## Source data

Source Data Figs. 2–6 and Extended Data Figs. 2, 4–8 Statistical source data for all figures and extended data figures in one workbook with separate tabs.

## Data Availability

All data are available in the Article or its Supplementary Information. Source data are provided with this paper.
