# Supplementary material for: RETICULATA1 is a plastid-localized basic amino acid transporter
Source: Nat Plants. 2025 Aug 22;11(9):1890–902. doi: 10.1038/s41477-025-02080-z (PMC12449267; doi:10.1038/s41477-025-02080-z)
Supplement: Supplementary file 1 — Supplementary Text, Supplementary Fig. 1 and descriptions for Supplementary Tables 1–9. [file 41477_2025_2080_MOESM1_ESM.pdf]

nature plants

Supplementary information https://doi.org/10.1038/s41477-025-02080-z

RETICULATA1 is a plastid-localized basic amino acid transporter

In the format provided by the authors and unedited

Supplementary information

Supplementary Text

RE proteins are unique to plastid-containing organisms

Orthologues of RE are found in all three branches of Archaeplastida (Extended Data Fig. 1a), i.e., the Rhodophyta (red algae), Glaucophyta, and Viridiplantae (green algae and land plants). The Archaeplastida plastids originate from a single primary endosymbiotic event[63], suggesting that RE proteins evolved early during the establishment of endosymbiosis. Notably, the genome of *Paulinella chromatophora,* which acquired its chromatophore through a distinct primary endosymbiosis event[63], also encodes a RE-related protein. Additionally, various organisms with complex plastids, acquired through secondary endosymbiosis, such as *Guillardia theta*, also possess orthologues of RE (Extended Data Fig. 1a). These findings indicate that RE proteins are essential for the function of plastids and the establishment of endosymbiosis.

RE1 overexpression in the re-6 mutant background complements the reticulate leaf phenotype of re-6

To unambiguously assign the mutant phenotype of *re-6*, complementation lines overexpressing *RE1* in the *re-6* mutant background under the control of a *UBIQUITIN10* promoter were created. Therefore, the *RE1* sequence was 'N-terminally' fused with a GFP sequence between the sequence of the predicted plastid transit peptide and that of the mature protein. The lines showed an average increase of the *RE1* transcript of 129-fold (*re-6* OEX1) and 454-fold (*re-6* OEX2) in seedlings, respectively (Extended Data Fig. 2c). Lines overexpressing *RE1* C-terminally fused to a GFP sequence did not complement the reticulate leaf phenotype (Extended Data Fig. 2a). Interestingly, the *lcd1-1* mutant which carries a single nucleotide C-to-T transition in the last codon of the *RE1* sequence (**C**AG to **T**AG), resulting in a change from Gln to a premature stop codon, displays a reticulate leaf phenotype[64]. These findings suggest that the terminal amino acid residue in the mature RE1 protein is crucial for its function.

To confirm the spatial expression pattern of *RE1* and *RER1* in the wildtype, mutants, and *RE1* OEX lines, total RNA was isolated from seedlings, rosettes, and roots. Mean normalized expression of *RE1* and *RER1* was analyzed via quantitative real time PCR. As shown previously, *RE1* and *RER1* expression is not restricted to leaves (Extended Data Fig. 2f-h)[23,25,27]. The expression of both genes is higher in rosette leaves compared to seedlings (Extended Data Fig. 2f,g). *RE1* expression is higher in seedlings compared to root tissue, indicating a predominant function in photosynthetic active tissues (Extended Data Fig. 2h).

The RE1 protein forms a homomeric complex in vivo

To further assess the subcellular localization of RE1, we analyzed available proteomic datasets with a focus on chloroplast envelopes. RE1 was detected in the envelope fraction of isolated plastids in Arabidopsis and pea[29,65-67]. Using enrichment factors, Bouchnak and colleagues could genuinely distinguish chloroplast inner envelope from outer envelope proteins and other contaminants. RE1 and RER1 were among the list of genuine proteins identified in the inner envelope of Arabidopsis chloroplasts[29]. Furthermore, a protease protection assay using isolated chloroplasts from wildtype and *re-6* plants supported an inner envelope localization of RE1. Following treatment with thermolysin and trypsin, a band corresponding to RE1 was protected from thermolysin digestion but became susceptible to subsequent trypsin treatment, consistent with an inner-envelope localization of the protein (Supplementary Fig. 1a, b). However, immunoblot analysis also revealed a non-specific band

present in both wildtype and mutant samples (Supplementary Fig. 1c). This non-specific signal is likely chloroplast-derived, as it was enriched in isolated chloroplasts compared to total leaf extracts and increased in intensity upon protease treatment. The inner-envelope localization of RE1 is supported by the presence of a plastid transit peptide at the N-terminus of both, RE1 and RER1 (Fig. 1c, d)[28].

In BN-PAGE analysis of wildtype chloroplasts, the RE1 protein was primarily detected in fractions corresponding to an apparent molecular mass range of approximately 70 kDa to 300 kDa, indicating that RE/RER proteins form stable, high molecular weight complexes *in vivo*[31]. To independently assess the functional assembly of RE/RER1 in an oligomeric state, we conducted immunoblot analysis of BN-PAGE on chloroplasts isolated from both wildtype and *re-6* mutant rosette leaves. The immunoblots, decorated with an antiserum specific to a peptide-epitope of RE1, revealed the accumulation of RE1 protein in a high molecular weight complex around 250 kDa, indicating the presence of a RE1 homohexamer. Importantly, this complex was exclusively observed in isolated chloroplasts from the wildtype but not the *re-6* mutant (Extended Data Fig. 9a,b). To explore the structural arrangement of the complex, we also generated a tentative AlphaFold 3[24] model of a RE1 homohexamer, which supports the potential for hexameric assembly, albeit with limited confidence (ipTM = 0.2; Extended Data Fig. 9c, d).

Yeast complementation and transport assays

To assess whether RE1 and/or RER1 can restore the growth phenotype of the *arg11* yeast mutant, we cloned the coding sequence of RE1 and RER1, excluding the predicted plastid transit peptide, into the yeast vector pDR195 under control of the constitutive *PMA1* promoter. To direct the RE1 and RER1 proteins to mitochondria in yeast, we fused them with the mitochondrial transit peptide from the *S. cerevisiae* cytochrome c oxidase (ScCOXIV) at their N-termini[51]. Constructs were generated with and without a N-terminal 6xHis tag, as C-terminal tagging interferes with the function of the RE1 protein (Supplementary Fig. 2a). Both proteins, with and without the N-terminal His-tag, complement the *arg11* yeast mutant phenotype when expressed from the *PMA1* promoter, indicating a N-terminal fusion does not interfere with the function of the proteins (Extended Data Fig. 4l,m). The Arabidopsis mitochondrial basic amino acid carrier AtmBAC1 served as additional positive control[21].

To functional assess the transport properties of isolated mitochondrial membranes carrying the RE1 protein, we conducted independent mitochondria isolations of the *arg11* yeast mutant transformed with the empty vector pDR195 (control) or the pDR195 vector expressing the RE1 protein under the control of the *PMA1* promoter. Isolated mitochondria were ruptured, membranes were enriched and reconstituted into lipid vesicles with or without preloading of 20 mM substrate via the freeze-thaw method[68]. External substrate was removed via size-exclusion chromatography and the transport was initiated by diluting the liposomes with transport mix containing 0.2 mM Orn and ^{14}C-Orn. This artificially applied, outward facing concentration gradient over the liposomal membrane leads to the export of 'cold' substrate from the liposomes, and simultaneously facilitates the uptake of radioactive labeled substrate into the liposomes, only if the respective transporter is capable of shuttling the selected substrate (Extended Data Fig. 5a)[69]. Using that method, we were able to measure the exchange activity of preloaded Orn against radioactive labeled Orn of reconstituted mitochondrial membranes carrying the RE1 protein from five independent mitochondria isolations (Extended Data Fig. 5b-e).

Additionally, we aimed to functional characterize the RE1 protein in more detail. Therefore, we generated expression constructs for different heterologous expression systems, including *E. coli*, cell-free *E. coli*, *S. cerevisiae* (driven by the strong galactose-inducible promoter), wheat germ cell-free, and Xenopus oocytes. We observed overexpression in Xenopus oocytes but no activity could be detected. Overexpression in *E. coli* yielded only little RE1 protein, which did not show transport activity. Cell-free expression in the *E. coli* and wheat germ system, as well as overexpression in yeast without target peptide with the galactose-inducible

system led to a high amount of RE1 protein. However, the protein did not show any transport activity when reconstituted. Our *in vivo* data suggests that RE1 functions as a homohexameric complex in chloroplasts (Supplementary Fig. 9a,b)[31]. We hypothesize that functional complex assembly, which is essential for transport activity, does not occur in the heterologous expression systems tested, leading to the absence of transport function.

Supplementary Figures

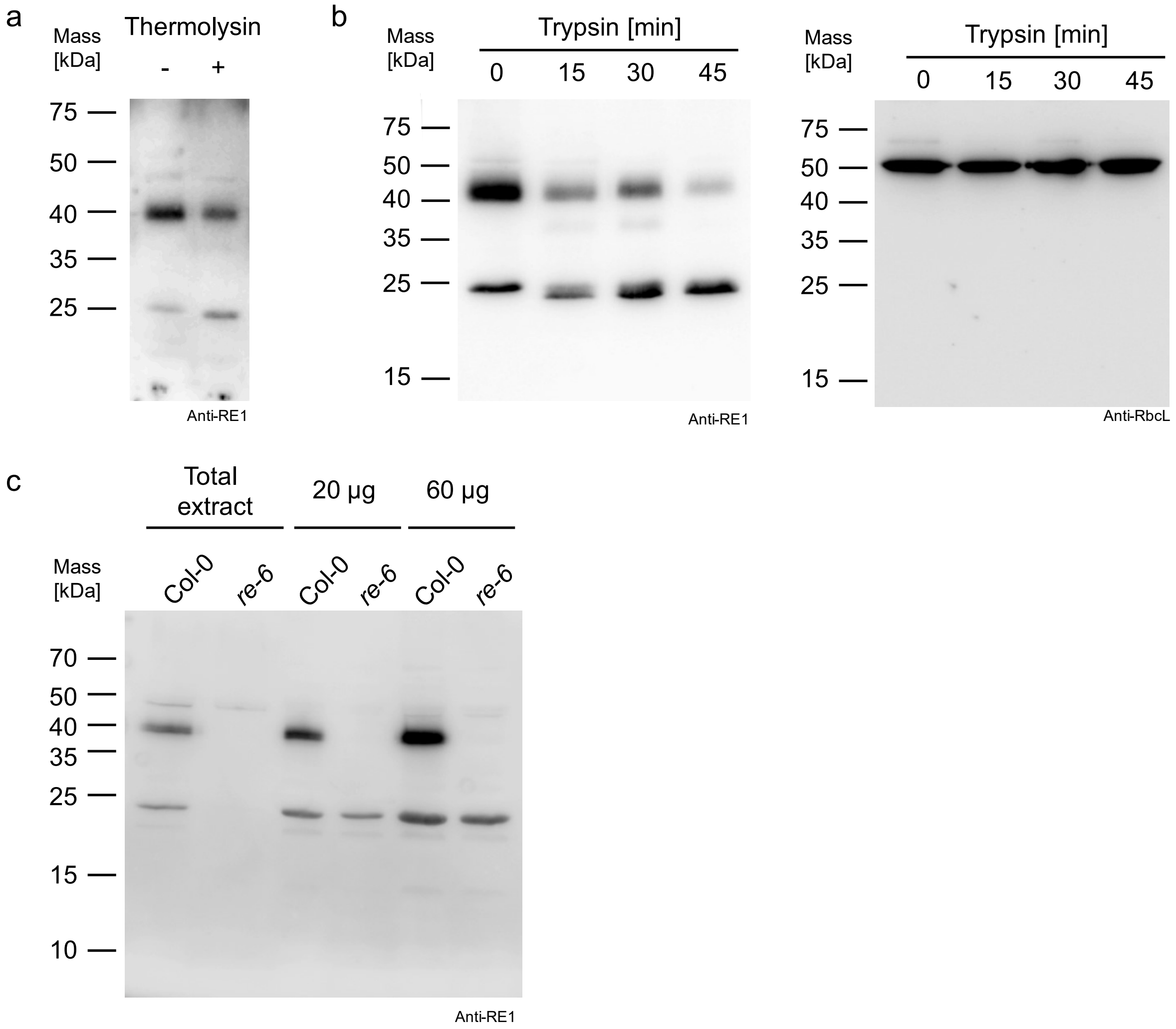

Supplementary Figure 1: Immunoblot analyses of isolated wildtype and *re-6* chloroplasts. (**a**) Isolated, intact chloroplasts from wildtype plants were treated for 30 min with thermolysin and subsequently analyzed by SDS-PAGE and immunoblotting. (**b**) Isolated, intact chloroplasts from wildtype plants were treated for 0, 15, 30, and 45 min with trypsin and subsequently analyzed by SDS-PAGE and immunoblotting. (**c**) Immunoblot analysis of total leaf extract, and isolated chloroplasts from wildtype and *re-6* plants. RE1 protein was visualized by immunodetection with an antibody raised against the RE1 protein. Large subunit of Rubsico (RbcL) was visualized immunodetection with an antibody raised against the RbcL protein.The experiment was independently repeated three times, with consistent results.

Supplementary Tables

Supplementary Table 1. Co-expression analysis of the gene encoding for aspartate kinase-homoserine dehydrogenase II (AT5G14060).

Top 50 co-expressed genes generated by ATTED-II using the default ath-u.3 settings[70]. Supportability is defined as the *p*-value threshold.

Supplementary Table 2. Co-expression analysis of the gene encoding for aspartate kinase 2 (AT4G19710).

Top 50 co-expressed genes generated by ATTED-II using the default ath-u.3 settings[70]. Supportability is defined as the *p*-value threshold.

Supplementary Table 3. Co-expression analysis of the gene encoding for RE1 (AT2G37860).

Top 50 co-expressed genes generated by ATTED-II using the default ath-u.3 settings[70]. Supportability is defined as the *p*-value threshold.

Supplementary Table 4. Relative amino acid content per mg fresh weight of wildtype, *RE1* and *RER1* knockout seedlings, and the *RE1* OEX lines.

Samples were harvested in the middle of the light period. Amino acids were quantified via hydrophilic interaction liquid chromatography coupled to mass spectrometry. Data were acquired and assessed as described in the Methods section and are shown as mean ± SD of four biological replicates. Different letters indicate statistically significant differences between means ($P < 0.05$; one-way ANOVA with Tukey's test).

Supplementary Table 5. Relative amino acid content per mg fresh weight of wildtype, *RE1* and *RER1* knockout rosettes, and the *RE1* OEX lines.

Samples were harvested in the middle of the light period. Amino acids were quantified via hydrophilic interaction liquid chromatography coupled to mass spectrometry. Data were acquired and assessed as described in the Methods section and are shown as mean ± SD of four biological replicates. Different letters indicate statistically significant differences between means ($P < 0.05$; one-way ANOVA with Tukey's test).

Supplementary Table 6. Relative amino acid content in isolated chloroplasts of Col-0 and *re-6*.

Chloroplasts were isolated from 3-week-old Col-0 and *re-6* plants. Amino acids were quantified via hydrophilic interaction liquid chromatography coupled to mass spectrometry. Data were acquired and assessed as described in the Methods section and are shown as mean ± SD of six biological replicates. Different letters indicate statistically significant differences between means ($P < 0.05$; one-way ANOVA with Tukey's test).

Supplementary Table 7. Lys treatment data set.

Relative responses from $^{15}NH_4Cl$ labeling experiment with or without 1 mM Lys. Data were acquired and assessed as described in the Methods section. Isotopologues are described as follows: unlabeled, $[M]^+$; labeled, $[M+N]^+$ with N corresponding to the number of labeled nitrogen atoms in the metabolite. Letters are used to distinguish between positional isomers e.g. $[M+1b]^+$.

Supplementary Table 8. Citr treatment data set.

Relative responses from $^{15}NH_4Cl$ labeling experiment with or without 5 mM Citr. Data were acquired and assessed as described in the Methods section. Isotopologues are described as follows: unlabeled, $[M]^+$; labeled, $[M+N]^+$ with N corresponding to the number of labeled nitrogen atoms in the metabolite. Letters are used to distinguish between positional isomers e.g. $[M+1b]^+$.

Supplementary Table 9. Oligonucleotides and guide sequences used in this study.
