# Supplementary material for: RETICULATA1 is a plastid-localized basic amino acid transporter
Source: Nat Plants. 2025 Aug 22;11(9):1890–902. doi: 10.1038/s41477-025-02080-z (PMC12449267; doi:10.1038/s41477-025-02080-z)
Supplement: Supplementary file 2 — Reporting Summary [file 41477_2025_2080_MOESM2_ESM.pdf]

nature portfolio

Corresponding author(s): Andreas Weber

Last updated by author(s): Jul 7, 2025

Reporting Summary

Nature Portfolio wishes to improve the reproducibility of the work that we publish. This form provides structure for consistency and transparency in reporting. For further information on Nature Portfolio policies, see our Editorial Policies and the Editorial Policy Checklist.

Statistics

For all statistical analyses, confirm that the following items are present in the figure legend, table legend, main text, or Methods section.

n/a	Confirmed	
☐	☒	The exact sample size (*n*) for each experimental group/condition, given as a discrete number and unit of measurement
☐	☒	A statement on whether measurements were taken from distinct samples or whether the same sample was measured repeatedly
☐	☒	The statistical test(s) used AND whether they are one- or two-sided<br>*Only common tests should be described solely by name; describe more complex techniques in the Methods section.*
☒	☐	A description of all covariates tested
☐	☒	A description of any assumptions or corrections, such as tests of normality and adjustment for multiple comparisons
☐	☒	A full description of the statistical parameters including central tendency (e.g. means) or other basic estimates (e.g. regression coefficient) AND variation (e.g. standard deviation) or associated estimates of uncertainty (e.g. confidence intervals)
☐	☒	For null hypothesis testing, the test statistic (e.g. *F*, *t*, *r*) with confidence intervals, effect sizes, degrees of freedom and *P* value noted<br>*Give P values as exact values whenever suitable.*
☒	☐	For Bayesian analysis, information on the choice of priors and Markov chain Monte Carlo settings
☒	☐	For hierarchical and complex designs, identification of the appropriate level for tests and full reporting of outcomes
☒	☐	Estimates of effect sizes (e.g. Cohen's *d*, Pearson's *r*), indicating how they were calculated

Our web collection on statistics for biologists contains articles on many of the points above.

Software and code

Policy information about availability of computer code

Data collection	StepOnePlus™ Real-Time PCR thermocycle from Applied Biosystems; Leica SP8 confocal microscope with Leica LAS X software; Tri-Carb® 4910 TR from PerkinElmer; Agilent MassHunter Workstation Software (B07.00); Thermo Scientific Chromeleon 7.2.9
Data analysis	R (version 4.3.1); ImageJ (version 1.54); Fiji (version 2.9) MAFFT (version 7); iTOL (version 7); Synthego ICE Analysis tool (version 3); Microsoft Excel 365; Skyline software (version 24.1.0.199); Agilent MassHunter Quantitative Analysis (B09.00)

For manuscripts utilizing custom algorithms or software that are central to the research but not yet described in published literature, software must be made available to editors and reviewers. We strongly encourage code deposition in a community repository (e.g. GitHub). See the Nature Portfolio guidelines for submitting code & software for further information.

Data

Policy information about availability of data

All manuscripts must include a data availability statement. This statement should provide the following information, where applicable:

- Accession codes, unique identifiers, or web links for publicly available datasets
- A description of any restrictions on data availability
- For clinical datasets or third party data, please ensure that the statement adheres to our policy

All data are available in the main text and/or the extended/supplementary information.

Research involving human participants, their data, or biological material

Policy information about studies with human participants or human data. See also policy information about sex, gender (identity/presentation), and sexual orientation and race, ethnicity and racism.

Reporting on sex and gender	*Use the terms sex (biological attribute) and gender (shaped by social and cultural circumstances) carefully in order to avoid confusing both terms. Indicate if findings apply to only one sex or gender; describe whether sex and gender were considered in study design; whether sex and/or gender was determined based on self-reporting or assigned and methods used. Provide in the source data disaggregated sex and gender data, where this information has been collected, and if consent has been obtained for sharing of individual-level data; provide overall numbers in this Reporting Summary. Please state if this information has not been collected. Report sex- and gender-based analyses where performed, justify reasons for lack of sex- and gender-based analysis.*
Reporting on race, ethnicity, or other socially relevant groupings	*Please specify the socially constructed or socially relevant categorization variable(s) used in your manuscript and explain why they were used. Please note that such variables should not be used as proxies for other socially constructed/relevant variables (for example, race or ethnicity should not be used as a proxy for socioeconomic status). Provide clear definitions of the relevant terms used, how they were provided (by the participants/respondents, the researchers, or third parties), and the method(s) used to classify people into the different categories (e.g. self-report, census or administrative data, social media data, etc.) Please provide details about how you controlled for confounding variables in your analyses.*
Population characteristics	*Describe the covariate-relevant population characteristics of the human research participants (e.g. age, genotypic information, past and current diagnosis and treatment categories). If you filled out the behavioural & social sciences study design questions and have nothing to add here, write "See above."*
Recruitment	*Describe how participants were recruited. Outline any potential self-selection bias or other biases that may be present and how these are likely to impact results.*
Ethics oversight	*Identify the organization(s) that approved the study protocol.*

Note that full information on the approval of the study protocol must also be provided in the manuscript.

Field-specific reporting

Please select the one below that is the best fit for your research. If you are not sure, read the appropriate sections before making your selection.

☒ Life sciences ☐ Behavioural & social sciences ☐ Ecological, evolutionary & environmental sciences

For a reference copy of the document with all sections, see nature.com/documents/nr-reporting-summary-flat.pdf

Life sciences study design

All studies must disclose on these points even when the disclosure is negative.

Sample size	All plant-based experiments were performed on at least 3 biological replicates. For metabolite analyses on seedlings, seedlings from one plate were pooled and treated as one biological replicate. Experiments with technical replicates (transport assays) were repeated at least 3 times with independent reconstitution events (independent mitochondria isolations). The exact sample size number of each experiment is stated in the figure legends. Pictures shown in the study (i.e. images showing plant growth, phenotypes on MS plates, yeast growth, and confocal microscopy images) are representative pictures.
Data exclusions	No data were excluded from the analyses.
Replication	All metabolite analyses were performed in at least 4 biological replicates, and independently repeated. qRT-PCR experiments were performed on 3-4 biological replicates. Transport assays, yeast complementation, and growth on MS plates supplemented with various amino acids were repeated at least 3 times independently. Chloroplast isolations and BN-PAGE analyses were performed at least 3 times independently. Silique length and number of seeds per siliques was measured from at least 73 mature siliques.
Randomization	Plants were grown in growth cabinets in a randomized fashion. Samples for metabolite extractions were randomized during the extraction process.
Blinding	Blinding for plant work was not possible as the RE1 knockout lines exhibit a very prominent and distinctive reticulate leaf mutant phenotype. Samples for metabolite extractions and analysis were labeled with a 3 digit number for each biological replicate. Extraction and analysis was performed based on the 3 digit number code, and the samples were linked back to their genetic background after data acquisition.

Reporting for specific materials, systems and methods

We require information from authors about some types of materials, experimental systems and methods used in many studies. Here, indicate whether each material, system or method listed is relevant to your study. If you are not sure if a list item applies to your research, read the appropriate section before selecting a response.

Materials & experimental systems

n/a	Involved in the study	
☐	☒	Antibodies
☒	☐	Eukaryotic cell lines
☒	☐	Palaeontology and archaeology
☒	☐	Animals and other organisms
☒	☐	Clinical data
☐	☐	Dual use research of concern
☐	☒	Plants

Methods

n/a	Involved in the study	
☒	☐	ChIP-seq
☒	☐	Flow cytometry
☒	☐	MRI-based neuroimaging

Antibodies

Antibodies used

Anti-RE1 antibody (Agrisera) The anti-RE1 antibody was raised against amino acids 73 to 86 at the N-terminal part of the RE1 protein, the antibody was used unpurified. 1:2,000 dilution for immunoblot
anti-RbcL antibody (Agrisera, Cat. No. AS03 037), 1:5,000 dilution for immunoblot
goat anti-rabbit-horse-radish peroxidase antibody (Merck Millipore, Cat. No. 12-348), 1:2,000 dilution for immunoblot

Validation

The anti-RE1 antibody was validated on total plant extract and isolated chloroplasts from Arabidopsis thaliana.
The secondary goat anti-rabbit-horse-radish peroxidase antibody was used as described in the manufacturer's instructions supplied from Merck Millipore.

Dual use research of concern

Policy information about dual use research of concern

Hazards

Could the accidental, deliberate or reckless misuse of agents or technologies generated in the work, or the application of information presented in the manuscript, pose a threat to:

No	Yes	
☒	☐	Public health
☒	☐	National security
☒	☐	Crops and/or livestock
☒	☐	Ecosystems
☒	☐	Any other significant area

Experiments of concern

Does the work involve any of these experiments of concern:

No	Yes	
☒	☐	Demonstrate how to render a vaccine ineffective
☒	☐	Confer resistance to therapeutically useful antibiotics or antiviral agents
☒	☐	Enhance the virulence of a pathogen or render a nonpathogen virulent
☒	☐	Increase transmissibility of a pathogen
☒	☐	Alter the host range of a pathogen
☒	☐	Enable evasion of diagnostic/detection modalities
☒	☐	Enable the weaponization of a biological agent or toxin
☒	☐	Any other potentially harmful combination of experiments and agents

Plants

Seed stocks

re-6 (Salk_084529), rer1-1 (Salk_126363), rer1-2 (Salk_073984), rer1-3 (Salk_093173). Mutant seeds were obtained from the Nottingham Arabidopsis Stock Centre (NASC).

Novel plant genotypes

UBQ10-RE1(cTP)-GFP-RE1(mature) (OEX) was transformed into re-6 by using Agrobacterium tumefaciens (strain GV3103)-mediated floral-dip method. Positive transformants were selected based on kanamycin resistance and confirmed by qRT-PCR and visual complementation of the reticulate leaf mutant phenotype. T3 generation were used for experiments.
UBQ10-RER1(cTP)-GFP-RER1(mature) (OEX RER1) was transformed into re-6 by using Agrobacterium tumefaciens (strain GV3103)-mediated floral-dip method. Positive transformants were selected based on kanamycin resistance and confirmed by PCR and visual complementation of the reticulate leaf mutant phenotype.
UBQ10-RE1(gDNA)-GFP, UBQ10-RE1(CDS)-GFP and UBQ10-RE1(gDNA) were transformed into re-6 and Col-0 by using Agrobacterium tumefaciens (strain GV3103)-mediated floral-dip method. Positive transformants were selected based on kanamycin resistance, confirmed by PCR and analyzed for complementation or non-complementation of the reticulate leaf mutant phenotype.
RER1 (AT5G22790) knockout mutants in the re-6 mutant background were generated using CRISPR/Cas9. Guides targeting the coding region of RER1 are listed in supplementary Table 9.

Authentication

The T-DNA insertion lines were verified by PCR using gene-specific primer pairs and the left border primer of the T-DNA insertion (supplemental table 9).
The generated CRISPR/Cas9 lines were verified by PCR using a gene-specific primer pair covering the area within the gene which was targeted by the selected guides. The PCR was purified and send for sequencing to validate events induced by Cas9.
